# Synthesis, biological evaluation, and molecular modelling studies of potent human neutrophil elastase (HNE) inhibitors

**DOI:** 10.1080/14756366.2018.1480615

**Published:** 2018-07-04

**Authors:** Maria Paola Giovannoni, Igor A. Schepetkin, Mark T. Quinn, Niccolò Cantini, Letizia Crocetti, Gabriella Guerrini, Antonella Iacovone, Paola Paoli, Patrizia Rossi, Gianluca Bartolucci, Marta Menicatti, Claudia Vergelli

**Affiliations:** a NEUROFARBA, Pharmaceutical and Nutraceutical Section, University of Florence, Sesto Fiorentino, Italy;; b Department of Microbiology and Immunology, Montana State University, Bozeman, MT, USA;; c Department of Industrial Engineering, University of Florence, Florence, Italy

**Keywords:** Isoxazol-5(2H)-one, HNE, molecular modelling, stability

## Abstract

We report the synthesis and biological evaluation of a new series of 3- or 4-(substituted)phenylisoxazolones as HNE inhibitors. Due to tautomerism of the isoxazolone nucleus, two isomers were obtained as final compounds (2-NCO and 5-OCO) and the 2-NCO derivatives were the most potent with IC_50_ values in the nanomolar range (20–70 nM). Kinetic experiments indicated that 2-NCO **7d** and 5-OCO **8d** are both competitive HNE inhibitors. Molecular modelling on **7d** and **8d** suggests for the latter a more crowded region about the site of the nucleophilic attack, which could explain its lowered activity. In addition molecular dynamics (MD) simulations showed that the isomer **8d** appears more prone to form H-bond interactions which, however, keep the reactive sites quite distant for the attack by Ser195. By contrast the amide **7d** appears more mobile within the active pocket, since it makes single H-bond interactions affording a favourable orientation for the nucleophilic attack.

## Introduction

Proteases are enzymes implicated in cellular reactions involving the cleavage of protein substrates^1^. Serine proteases are characterised by the presence of a serine residue at the active site^2^. They are divided into four classes: chymotrypsin, subtilisin, carboxypeptidase Y, and caseinolytic protease^3^. Human neutrophil elastase (HNE), proteinase 3 (PR3), cathepsin G, and the recently discovered NSP4^4^ are serine proteases belonging to the chymotrypsin family and represent neutrophil serine proteases (NSP)^5^^,^^6^. NSP are synthesised and expressed in neutrophil azurophilic granules. Neutrophils play a pivotal role in host defence, inflammation and tissue remodelling, and HNE is a key mediator of neutrophil-driven inflammation^7^.

HNE is a small, basic, and soluble glycoprotein of about 30 kDa^8^ that performs many functions in our body. For example, HNE is involved in the maintenance of tissue homeostasis; it degrades a variety of structural proteins of the extracellular matrix, such as elastin, fibronectin, collagen, proteoglycan, and laminin; and it repairs damaged tissue^9^. Moreover, HNE plays an important and dual role in inflammation by degrading pro-inflammatory cytokines to reduce the intensity of the inflammation but also increasing the secretion of pro-inflammatory factors^10^. In the case of infection, HNE plays as an intracellular function in destroying phagocytosed pathogens, as well as an extracellular function through the formation of neutrophil extracellular traps, which can trap and kill microorganisms^11^^,^^12^. The powerful HNE activity is tightly controlled by the presence of extracellular neutralizing endogenous serine protease inhibitors, such as α-1 antitrypsin, secretory leucocyte protease inhibitor (SLPI), and elafin^13^. α1-Antitrypsin is a water-soluble glycoprotein classified as systemic HNE inhibitor that is synthesised in the liver and is particularly abundant in the lungs^14^. In contrast, SLPI and elafin are classified as alarm inhibitors because they are produced and released directly into the airway epithelium in response to the release of cytokines, regulating the immune response and inflammatory processes^15–17^. Under physiological conditions, the balance between protease and anti-protease supports the maintenance of tissue homeostasis. However, if this balance fails, excessive HNE activity can cause tissue the damage associated with some serious chronic diseases^4^^,^^6^. Among the pathologies associated with increased HNE activity are adult respiratory distress syndrome (ARDS)^18^, chronic obstructive pulmonary disease (COPD)^19^^,^^20^, cystic fibrosis (CF)^21^^,^^22^, and other disorders with an inflammatory component, such as rheumatoid arthritis^23^, atherosclerosis^24^, psoriasis, and dermatitis^25^. Recently, it was demonstrated that HNE is also implicated in the progression of non-small cell lung cancer^26^.

**Figure 1.**
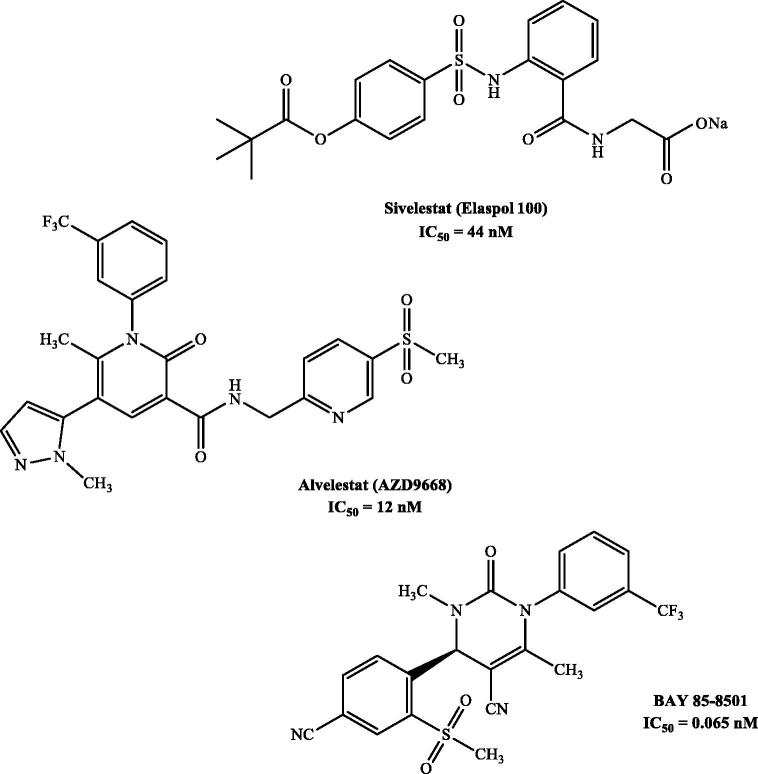
Potent HNE inhibitors.

Although a large number of molecules have been reported as HNE inhibitors^27^^,^^28^, only two drugs are currently available for clinical use: Prolastin (purified α1-AT), a peptide drug synthesised by recombinant DNA techniques^29^ and used for the treatment of α1-antitripsin deficiency (AATD)^30^, and Sivelestat (Elaspol^®^100), a non-peptide low molecular weight compound, belonging to the second generation of HNE inhibitors^31^. Sivelestat has an IC_50_ = 44 nM and is currently marketed only in Japan and South Korea^32^^,^^33^ (Figure 1). AZD9668 (Alvelestat, AstraZeneca)^34^ and BAY 85-8501 (Bayer HealthCare)^35^ are two potent HNE inhibitors, belonging to the third and fifth generations of HNE inhibitors^31^, respectively, that have recently reached Phase II of clinical trials for COPD, CF, and BE (Figure 1).

In recent research focused on the development of new HNE inhibitors, we investigated various bicyclic scaffolds, such as indazole^36^^,^^37^, indole^38^, and cinnolinone^39^, compounds. The most interesting inhibitors were effective in the nanomolar range, with a potency comparable to Sivelestat. Subsequently, we focused our research on the design and synthesis of monocyclic derivatives with an isoxazol-*5*(*2H*)-one core, and the first series of 3/4-alkyl-(di)substituted isoxazolones was recently published^40^. We report here the synthesis of a new series of isoxazolone derivatives bearing a (substituted)phenyl at positions *3* and *4* and biological evaluation of their HNE inhibitory activity.

## Material and methods

All melting points were determined on a Büchi apparatus (New Castle, DE) and are uncorrected. Extracts were dried over Na_2_SO_4_, and the solvents were removed under reduced pressure. Merck F-254 commercial plates (Merck, Durham, NC) were used for analytical TLC to follow the course of reactions. Silica gel 60 (Merck 70–230 mesh, Merck, Durham, NC) was used for column chromatography. ^1^H NMR, ^13^C NMR, HSQC, HMBC, and NOESY bidimensional spectra were recorded on an Avance 400 instrument (Bruker Biospin Version 002 with SGU, Bruker Inc., Billerica, MA). Chemical shifts (*δ*) are reported in ppm to the nearest 0.01 ppm using the solvent as an internal standard. Coupling constants (*J* values) are given in Hz and were calculated using TopSpin 1.3 software (Nicolet Instrument Corp., Madison, WI) and are rounded to the nearest 0.1 vHz. Mass spectra (*m*/*z*) were recorded on an ESI-TOF mass spectrometer (Brucker Micro TOF, Bruker Inc., Billerica, MA), and reported mass values are within the error limits of ± 5 ppm mass units. Microanalyses indicated by the symbols of the elements or functions were performed with a Perkin–Elmer 260 elemental analyser (PerkinElmer, Inc., Waltham, MA) for C, H, and N, and the results were within ± 0.4% of the theoretical values, unless otherwise stated. Reagents and starting material were commercially available.

### Chemistry

#### 3-Methyl-2-(3-methylbenzyl)-4-phenylisoxazol-5(2H)-one (2)

A mixture of the appropriate intermediate (**1a**^41^) (0.57 mmol), K_2_CO_3_ (1.14 mmol), and 1-(chloromethyl)-3-methylbenzene (0.86 mmol) in 2 ml of anhydrous acetonitrile was stirred at reflux for 2 h. After cooling, the mixture was concentrated *in vacuo*, diluted with ice-cold water (10 ml), and extracted with ethyl acetate (3 × 15 ml). The organic phase was dried over sodium sulphate and the solvent was evaporated *in vacuo* to afford the final compound **2**, which was purified by column chromatography using cyclohexane/ethyl acetate 2:1 as eluent. Yield =57%; oil. ^1^H NMR (CDCl_3_-d_1_) *δ* 2.35 (s, 6H, 2 × CH_3_), 4.79 (s, 2H, CH_2_), 7.07–7.14 (m, 3H, Ar), 7.22–7.28 (m, 2H, Ar), 7.38 (t, 2H, Ar, *J =* 7.8 Hz), 7.45 (d, 2H, Ar, *J =* 7.2 Hz). ^13^C NMR (CDCl_3_-d_1_) *δ* 12.44 (CH_3_), 21.63 (CH_3_), 54.90 (CH_2_), 103.58 (C), 125.16 (CH), 127.17 (CH), 128.21 (CH), 128.58 (CH), 128.82 (CH), 128.90 (CH), 129.40 (CH), 129.77 (C), 133.45 (C), 138.75 (C), 158.65 (C), 169.53 (C). ESI-MS calcd. for C_18_H_17_NO_2_, 279.33; found: *m/z* 280.13 [M + H]^+^. Anal. C_18_H_17_NO_2_ (C, H, N).

#### General procedure for compounds (3a–c)

To a suspension of the appropriate 4-substituted benzensulfonyl chloride (0.16 mmol) in 3 ml of anhydrous pyridine, 0.79 mmol of intermediate **1a**^41^ was added. The mixture was stirred at room temperature for 4 h. The solvent was concentrated *in vacuo* to afford the final compounds **3a–c** which were purified by column chromatography using cyclohexane/ethyl acetate in different ratio (2:1 for **3a**, 4:1 for **3b**) or toluene/ethyl acetate 9:1 for **3c** as eluents.

##### 2-((4-Hydroxyphenyl)sulfonyl)-3-methyl-4-phenylisoxazol-5(2H)-one (3a)

Yield* =* 30%; mp* =* 50–51 °C (EtOH). ^1^H NMR (CDCl_3_-d_1_) *δ* 2.57 (s, 3H, CH_3_), 6.93 (d, 2H, Ar, *J =* 8.8 Hz), 7.30–7.40 (m, 5H, Ar), 7.78 (d, 2H, Ar, *J =* 8.8 Hz). ^13^C NMR (CDCl_3_-d_1_) *δ* 14.59 (CH_3_), 113.94 (C), 116.39 (CH), 121.92 (C), 126.91 (C), 128.77 (CH), 128.87 (CH), 129.19 (CH), 131.88 (CH), 158.94 (C), 162.88 (C), 168.80 (C). ESI-MS calcd. for C_16_H_13_NO_5_S, 331.34; found: *m/z* 332.05 [M + H]^+^. Anal. C_16_H_13_NO_5_S (C, H, N).

##### 4-((3-Methyl-5-oxo-4-phenylisoxazol-2(5H)-yl)sulfonyl)phenyl pivalate (3b)

Yield* =* 15%; mp* =* 115–116 °C (EtOH). ^1^H NMR (CDCl_3_-d_1_) *δ* 1.35 (s, 9H, C(CH_3_)_3_), 2.58 (s, 3H, CH_3_), 7.26 (d, 2H, Ar, *J =* 8.0 Hz), 7.32 (d, 2H, Ar, *J =* 8.6 Hz), 7.35–7.40 (m, 3H, Ar), 7.95 (d, 2H, Ar, *J =* 8.6 Hz). ^13^C NMR (CDCl_3_-d_1_) *δ* 14.50 (CH_3_), 26.97 (CH_3_), 29.37 (C), 39.36 (C), 114.27 (C), 122.81 (CH), 127.03 (C), 128.45 (CH), 128.81 (CH), 129.11 (CH), 130.39 (CH), 130.97 (CH), 156.83 (C), 157.77 (C), 167.45 (C), 175.75 (C). ESI-MS calcd. for C_21_H_21_NO_6_S, 415.46; found: *m/z* 416.11 [M + H]^+^. Anal. C_21_H_21_NO_6_S (C, H, N).

##### N-(4-((3-methyl-5-oxo-4-phenylisoxazol-2(5H)-yl)sulfonyl)phenyl)pivalamide (3c)

Yield* =* 72%; mp* =* 153–155 °C (EtOH). ^1^H NMR (CDCl_3_-d_1_) *δ* 1.30 (s, 9H, C(CH_3_)_3_), 2.58 (s, 3H, CH_3_), 7.26 (d, 1H, Ar, *J =* 6.8 Hz), 7.36–7.42 (m, 4H, Ar), 7.70 (exch br s, 1H, NH), 7.79 (d, 2H, Ar, *J =* 8.8 Hz), 7.84 (d, 2H, Ar, *J =* 8.8 Hz). ^13^C NMR (CDCl_3_-d_1_) *δ* 14.61 (CH_3_), 27.42 (CH_3_), 44.65 (C), 113.95 (C), 119.39 (CH), 125.36 (C), 125.85 (CH), 128.43 (CH), 128.81 (CH), 128.97 (CH), 129.88 (CH), 130.68 (CH), 130.91 (CH), 134.00 (C), 144.98 (C), 158.06 (C), 165.00 (C), 177.20 (C). ESI-MS calcd. for C_21_H_22_N_2_O_5_S, 414.47; found: *m/z* 415.13 [M + H]^+^. Anal. C_21_H_22_N_2_O_5_S (C, H, N).

#### General procedure for compounds (4a–h, 4n–t)

To a suspension of the appropriate substrates **1a–e** (**1a**^41^, **1b**^42^, **1c,d**^43^, and **1e**^44^) (0.86 mmol) in 10 ml of anhydrous THF, 1.72 mmol of sodium hydride (60% dispersion in mineral oil), and 1.03 mmol of the appropriate acyl/aroyl chloride were added. The mixture was stirred at room temperature overnight. The solvent was concentrated *in vacuo* to obtain the final compounds **4a–h** and **4n–t** which were purified by column chromatography using hexane/ethyl acetate (5:1 for **4a**,**c**,**d**; 5:2 for **4e**,**g**), cyclohexane/ethyl acetate (1:1 for **4f**; 3:1 for **4t**; 4:1 for **4h**; 5:1 for **4o–s**; 6:1 for **4n**), or toluene/ethyl acetate 9.5:0.5 (for **4b**) as eluents.

##### 3-Methyl-2-(3-methylbenzoyl)-4-phenylisoxazol-5(2H)-one (4a)

Yield* =* 52%; mp* =* 85–88 °C (EtOH). ^1^H NMR (CDCl_3_-d_1_) *δ* 2.43 (s, 3H, m-*CH_3_*-Ph), 2.79 (s, 3H, CH_3_), 7.35–7.40 (m, 3H, Ar), 7.43–7.51 (m, 4H, Ar), 7.70–7.75 (m, 2H, Ar). ^13^C NMR (CDCl_3_-d_1_) *δ* 15.09 (CH_3_), 21.58 (CH_3_), 108.39 (C), 127.08 (CH), 127.62 (C), 128.28 (CH), 128.50 (CH), 128.81 (CH), 129.08 (CH), 130.28 (CH), 131.14 (C), 134.07 (CH), 138.32 (C), 154.62 (C), 163.79 (C), 165.90 (C). IR (*υ*)* =* 1690 cm^−1^ (CO amide), 1750 cm^−1^ (CO ester). ESI-MS calcd. for C_18_H_15_NO_3_, 293.32; found: *m/z* 294.11 [M + H]^+^. Anal. C_18_H_15_NO_3_ (C, H, N).

##### 2-(Cyclopropanecarbonyl)-3-methyl-4-phenylisoxazol-5(2H)-one (4b)

Yield* =* 63%; mp* =* 83–86 °C (EtOH). ^1^H NMR (DMSO-d_6_) *δ* 1.02–1.07 (m, 2H, CH_2_ cC_3_H_5_), 1.09–1.15 (m, 2H, CH_2_ cC_3_H_5_), 2.36–2.41 (m, 1H, CH cC_3_H_5_), 2.58 (s, 3H, CH_3_), 7.35–7.41 (m, 1H, Ar), 7.43–7.48 (m, 4H, Ar). ^13^C NMR (DMSO-d_6_) *δ* 10.82 (CH_2_), 13.25 (CH_3_), 15.14 (CH), 106.28 (C), 128.25 (C), 128.58 (CH), 129.10 (CH), 129.35 (CH), 154.69 (C), 166.05 (C), 169.02 (C). IR (υ)* =* 1695 cm^−1^ (CO amide), 1755 cm^−1^ (CO ester). ESI-MS calcd. for C_14_H_13_NO_3_, 243.26; found: *m/z* 244.09 [M + H]^+^. Anal. C_14_H_13_NO_3_ (C, H, N).

##### 3-Methyl-2-(4-methylbenzoyl)-4-phenylisoxazol-5(2H)-one (4c)

Yield* =* 35%; mp* =* 134–136 °C (EtOH). ^1^H NMR (CDCl_3_-d_1_) *δ* 2.51 (s, 3H, p-*CH_3_*-Ph), 2.87 (s, 3H, CH_3_), 7.38 (d, 2H, Ar, *J =* 8.0 Hz), 7.43–7.48 (m, 1H, Ar), 7.51–7.58 (m, 4H, Ar), 7.92 (d, 2H, Ar, *J =* 8.4 Hz). ^13^C NMR (CDCl_3_-d_1_) *δ* 15.37 (CH_3_), 22.02 (CH_3_), 108.19 (C), 127.85 (C), 128.46 (CH), 128.59 (CH), 128.95 (CH), 129.19 (CH), 128.19 (CH), 130.28 (CH), 144.55 (C), 154.60 (C), 158.00 (C), 163.62 (C), 166.13 (C). IR (υ)* =* 1672 cm^−1^ (CO amide), 1755 cm^−1^ (CO ester). ESI-MS calcd. for C_18_H_15_NO_3_, 293.32; found: *m/z* 294.11 [M + H]^+^. Anal. C_18_H_15_NO_3_ (C, H, N).

##### 3-Methyl-2-(2-methylbenzoyl)-4-phenylisoxazol-5(2H)-one (4d)

Yield* =* 42%; mp* =* 99–100 °C (EtOH). ^1^H NMR (CDCl_3_-d_1_) *δ* 2.47 (s, 3H, o-*CH_3_*-Ph), 2.85 (s, 3H, CH_3_), 7.32 (d, 2H, Ar, *J =* 6.8 Hz), 7.40–7.49 (m, 7H, Ar). ^13^C NMR (CDCl_3_-d_1_) *δ* 15.37 (CH_3_), 20.22 (CH_3_), 108.95 (C), 126.27 (CH), 128.10 (C), 128.74 (CH), 129.14 (CH), 129.43 (CH), 129.60 (CH), 131.53 (CH), 132.10 (CH), 132.52 (C), 137.22 (C), 153.99 (C), 164.91 (C), 166.31 (C). IR (*υ*)* =* 1688 cm^−1^ (CO amide), 1767 cm^−1^ (CO ester). ESI-MS calcd. for C_18_H_15_NO_3_, 293.32; found: *m/z* 294.11 [M + H]^+^. Anal. C_18_H_15_NO_3_ (C, H, N).

##### 3-Methyl-4-phenyl-2-(3-(trifluoromethyl)benzoyl)isoxazol-5(2H)-one (4e)

Yield* =* 42%; mp* =* 96–97 °C (EtOH). ^1^H NMR (CDCl_3_-d_1_) *δ* 2.85 (s, 3H, CH_3_), 7.40–7.45 (m, 1H, Ar), 7.48–7.54 (m, 4H, Ar), 7.69 (t, 1H, Ar, *J =* 8.0 Hz), 7.90 (d, 1H, Ar, *J =* 7.8 Hz), 8.15 (d, 1H, Ar, *J =* 8.0 Hz), 8.20 (s, 1H, Ar). ^13^C NMR (CDCl_3_-d_1_) *δ* 15.61 (CH_3_), 109.53 (C), 125.39 (C), 127.34 (CH), 127.38 (C), 127.84 (CH), 129.33 (CH), 129.49 (CH), 129.63 (CH), 129.71 (CH), 130.28 (C), 131.69 (C), 132.75 (CH), 133.47 (CH), 154.88 (C), 162.74 (C), 166.07 (C). ^19^F NMR (CDCl_3_-d_1_) *δ* − 62.82. IR (*υ*)* =* 1689 cm^−1^ (CO amide), 1738 cm^−1^ (CO ester). ESI-MS calcd. for C_18_H_12_F_3_NO_3_, 347.29; found: *m/z* 348.08 [M + H]^+^. Anal. C_18_H_12_F_3_NO_3_ (C, H, N).

##### 3-Methyl-2-(3-(methylsulfonyl)benzoyl)-4-phenylisoxazol-5(2H)-one (4f)

Yield* =* 15%; mp* =* 185–186 °C (EtOH). ^1^H NMR (CDCl_3_-d_1_) *δ* 2.83 (s, 3H, CH_3_), 3.13 (s, 3H, *CH_3_*SO_2_), 7.40–7.45 (m, 1H, Ar), 7.48–7.55 (m, 4H, Ar), 7.75 (t, 1H, Ar, *J =* 8.0 Hz), 8.19 (d, 2H, Ar, *J =* 7.6 Hz), 8.46 (s, 1H, Ar). ^13^C NMR (CDCl_3_-d_1_) *δ* 14.98 (CH_3_), 44.52 (CH_3_), 109.12 (C), 127.11 (C), 128.84 (CH), 128.93 (CH), 129.02 (CH), 129.75 (CH), 131.47 (CH), 132.84 (C), 134.47 (CH), 141.35 (C), 154.22 (C), 161.55 (C), 165.36 (C). ESI-MS calcd. for C_18_H_15_NO_5_S, 357.38; found: *m/z* 358.07 [M + H]^+^. Anal. C_18_H_15_NO_5_S (C, H, N).

##### 3-(3-Methyl-5-oxo-4-phenyl-2,5-dihydroisoxazole-2-carbonyl)benzonitrile (4g)

Yield* =* 7%; mp* =* 118–119 °C (EtOH). ^1^H NMR (CDCl_3_-d_1_) *δ* 2.83 (s, 3H, CH_3_), 7.45–7.55 (m, 5H, Ar), 7.67 (t, 1H, Ar, *J* = 7.2 Hz), 7.89 (d, 1H, Ar, *J* = 6.4 Hz), 8.16 (d, 1H, Ar, *J* = 7.6 Hz), 8.20 (s, 1H, Ar). ^13^C NMR (CDCl_3_-d_1_) *δ* 14.94 (CH_3_), 107.22 (C), 112.76 (C), 118.63 (C), 127.98 (CH), 128.67 (CH), 128.91 (CH), 129.54 (CH), 130.71 (CH), 131.86 (CH), 134.54 (C), 134.95 (C), 135.62 (CH), 139.52 (C), 157.64 (C), 165.87 (C). ESI-MS calcd. for C_18_H_12_N_2_O_3_, 304.30; found: *m/z* 305.09 [M + H]^+^. Anal. C_18_H_12_N_2_O_3_ (C, H, N).

##### 4-(3-Methyl-5-oxo-4-phenyl-2,5-dihydroisoxazole-2-carbonyl)benzonitrile (4h)

Yield* =* 46%; mp* =* 178–180 °C dec. (EtOH). ^1^H NMR (CDCl_3_-d_1_) *δ* 2.82 (s, 3H, CH_3_), 7.37–7.42 (m, 1H, Ar), 7.45–7.50 (m, 4H, Ar), 7.80 (d, 2H, Ar, *J* = 8.4 Hz), 8.00 (d, 2H, Ar, *J* = 8.4 Hz). ^13^C NMR (CDCl_3_-d_1_) *δ* 14.94 (CH_3_), 109.39 (C), 116.49 (C), 117.69 (C), 127.10 (C), 128.85 (CH), 128.93 (CH), 129.03 (CH), 130.41 (CH), 132.32 (CH), 135.19 (C), 154.20 (C), 161.73 (C), 165.45 (C). ESI-MS calcd. for C_18_H_12_N_2_O_3_, 304.30; found: *m/z* 305.09 [M + H]^+^. Anal. C_18_H_12_N_2_O_3_ (C, H, N).

##### 2-(Cyclopropanecarbonyl)-3,4-diphenylisoxazol-5(2H)-one (4n)

Yield* =* 21%; mp* =* 100–103 °C (EtOH). ^1^H NMR (CDCl_3_-d_1_) *δ* 1.06–1.11 (m, 2H, CH_2_ cC_3_H_5_), 1.16–1.21 (m, 2H, CH_2_ cC_3_H_5_), 1.80–1.86 (m, 1H, CH cC_3_H_5_), 7.17–7.22 (m, 2H, Ar), 7.31–7.40 (m, 5H, Ar), 7.39 (d, 1H, Ar, *J =* 7.2 Hz), 7.46 (d, 2H, Ar, *J =* 7.6 Hz). ^13^C NMR (CDCl_3_-d_1_) *δ* 10.39 (CH_2_), 12.51 (CH), 103.59 (C), 128.03 (CH), 128.31 (CH), 128.37 (CH), 128.72 (CH), 129.16 (CH), 129.84 (CH), 134.92 (C), 142.60 (C), 170.20 (C), 180.73 (C). ESI-MS calcd. for C_19_H_15_NO_3_, 305.33; found: *m/z* 306.11 [M + H]^+^. Anal. C_19_H_15_NO_3_ (C, H, N).

##### 4-Methyl-2-(3-methylbenzoyl)-3-phenylisoxazol-5(2H)-one (4o)

Yield* =* 27%; oil. ^1^H NMR (CDCl_3_-d_1_) *δ* 1.96 (s, 3H, CH_3_), 2.40 (s, 3H, m-*CH_3_*-Ph), 7.32–7.40 (m, 2H, Ar), 7.44–7.50 (m, 5H, Ar), 7.70 (d, 2H, Ar, *J =* 6.8 Hz). ^13^C NMR (CDCl_3_-d_1_) *δ* 7.57 (CH_3_), 21.63 (CH_3_), 105.74 (C), 127.37 (CH), 128.01 (CH), 128.33 (CH), 128.67 (CH), 128.82 (C), 130.51 (CH), 130.60 (CH), 131.07 (C), 134.31 (CH), 138.46 (C), 156.85 (C), 165.24 (C), 169.02 (C). ESI-MS calcd. for C_18_H_15_NO_3_, 293.32; found: *m/z* 294.11 [M + H]^+^. Anal. C_18_H_15_NO_3_ (C, H, N).

##### 2-(Cyclopropanecarbonyl)-4-methyl-3-phenylisoxazol-5(2H)-one (4p)

Yield* =* 31%; oil. ^1^H NMR (CDCl_3_-d_1_) *δ* 1.04–1.09 (m, 2H, CH_2_ cC_3_H_5_), 1.10–1.15 (m, 2H, CH_2_ cC_3_H_5_), 1.86 (s, 3H, CH_3_), 2.38–2.44 (m, 1H, CH cC_3_H_5_), 7.36–7.41 (m, 2H, Ar), 7.43–7.48 (m, 3H, Ar). ^13^C NMR (CDCl_3_-d_1_) *δ* 7.30 (CH_3_), 10.55 (CH_2_), 12.71 (CH), 104.82 (C), 127.78 (C), 128.21 (CH), 128.35 (CH), 128.51 (CH), 130.44 (CH), 154.89 (C), 168.03 (C), 168.99 (C). ESI-MS calcd. for C_14_H_13_NO_3_, 243.26; found: *m/z* 244.09 [M + H]^+^. Anal. C_14_H_13_NO_3_ (C, H, N).

##### 2-(3-Methylbenzoyl)-3-phenylisoxazol-5(2H)-one (4q)

Yield* =* 72%; oil. ^1^H NMR (CDCl_3_-d_1_) *δ* 2.46 (s, 3H, m-*CH_3_*-Ph), 6.54 (s, 1H, CH), 7.43–7.50 (m, 5H, Ar), 7.82–7.87 (m, 2H, Ar), 8.11–8.16 (m, 2H, Ar). ^13^C NMR (CDCl_3_-d_1_) *δ* 21.24 (CH_3_), 85.88 (CH), 126.76 (CH), 126.98 (C), 127.94 (CH), 129.04 (CH), 129.18 (C), 130.38 (CH), 131.23 (CH), 135.69 (CH), 139.06 (C), 160.47 (C), 164.26 (C), 165.70 (C). IR (*υ*)* =* 1600 cm^−1^ (CO amide), 1757 cm^−1^ (CO ester). ESI-MS calcd. for C_17_H_13_NO_3_, 279.29; found: *m/z* 280.09 [M + H]^+^. Anal. C_17_H_13_NO_3_ (C, H, N).

##### 2-(Cyclopropanecarbonyl)-3-phenylisoxazol-5(2H)-one (4r)

Yield* =* 57%; oil. ^1^H NMR (CDCl_3_-d_1_) *δ* 1.10–1.16 (m, 2H, CH_2_ cC_3_H_5_), 1.23–1.28 (m, 2H, CH_2_ cC_3_H_5_), 1.84–1.90 (m, 1H, CH cC_3_H_5_), 6.34 (s, 1H, CH), 7.42–7.47 (m, 3H, Ar), 7.74–7.79 (m, 2H, Ar). ^13^C NMR (CDCl_3_-d_1_) *δ* 10.82 (CH_2_), 12.71 (CH), 85.75 (CH), 126.55 (CH), 128.87 (CH), 129.27 (C), 130.26 (CH), 164.15 (C), 165.51 (C), 168.48 (C). ESI-MS calcd. for C_13_H_11_NO_3_, 229.23; found: *m/z* 230.08 [M + H]^+^. Anal. C_13_H_11_NO_3_ (C, H, N).

##### 2-(3-Methylbenzoyl)-3-(4-nitrophenyl)isoxazol-5(2H)-one (4s)

Yield* =* 40%; mp* =* 177–180 °C dec. (EtOH). ^1^H NMR (DMSO-d_6_) *δ* 2.42 (s, 3H, m-*CH_3_*-Ph), 7.13 (s, 1H, CH), 7.54 (t, 1H, Ar, *J =* 7.6 Hz), 7.64 (d, 1H, Ar, *J =* 7.6 Hz), 7.95–8.00 (m, 2H, Ar), 8.20 (d, 2H, Ar, *J =* 8.8 Hz), 8.36 (d, 2H, Ar, *J =* 8.8 Hz). ^13^C NMR (DMSO-d_6_) *δ* 21.27 (CH_3_), 124.60 (CH), 125.76 (C), 126.91 (CH), 127.99 (CH), 128.28 (CH), 128.91 (CH), 130.18 (CH), 131.20 (C), 133.90 (CH), 136.50 (C), 138.35 (C), 148.54 (C), 160.45 (C), 167.84 (C). ESI-MS calcd. for C_17_H_12_N_2_O_5_, 324.29; found: *m/z* 325.08 [M + H]^+^. Anal. C_17_H_12_N_2_O_5_ (C, H, N).

##### 2-(Cyclopropanecarbonyl)-3-(4-nitrophenyl)isoxazol-5(2H)-one (4t)

Yield* =* 23%; mp* =* 160–163 °C (EtOH). ^1^H NMR (CDCl_3_-d_1_) *δ* 1.16–1.21 (m, 2H, CH_2_ cC_3_H_5_), 1.27–1.32 (m, 2H, CH_2_ cC_3_H_5_), 1.87–1.94 (m, 1H, CH cC_3_H_5_), 6.43 (s, 1H, CH), 7.96 (d, 2H, Ar, *J =* 8.8 Hz), 8.31 (d, 2H, Ar, *J =* 8.8 Hz). ^13^C NMR (CDCl_3_-d_1_) *δ* 10.55 (CH_2_), 12.71 (CH), 86.27 (CH), 121.42 (C), 124.67 (CH), 127.37 (CH), 135.22 (C), 148.74 (C), 162.53 (C), 168.75 (C). ESI-MS calcd. for C_13_H_10_N_2_O_5_, 274.23; found: *m/z* 275.06 [M + H]^+^. Anal. C_13_H_10_N_2_O_5_ (C, H, N).

#### General procedure for compounds (4i, 4l)

To a suspension of 4-(pivaloyoxy)benzoic acid^45^ or 4-(pivalamido)benzoic acid^46^ (0.32 mmol) in 1 ml of anhydrous toluene, 0.64 mmol of SOCl_2_, and a catalytic amount of DMF (0.05 mmol) were added. The mixture was stirred at reflux for 2 h. The solvent was concentrated *in vacuo* and the crude compound was used without purification and added to a previously prepared solution composed of 0.29 mmol of intermediate **1a**^41^ and 0.64 mmol of sodium hydride in 5 ml of anhydrous THF. The mixture was stirred at room temperature overnight. After evaporation of the solvent, the product was purified by column chromatography using toluene/ethyl acetate 9:1 for **4i** and hexane/acetone 4:1 for **4l** as eluents.

##### N-(4-(3-methyl-5-oxo-4-phenyl-2,5-dihydroisoxazole-2-carbonyl)phenyl)pivalamide (4i)

Yield* =* 10%; mp* =* 132–134 °C (EtOH). ^1^H NMR (CDCl_3_-d_1_) *δ* 1.37 (s, 9H, C(CH_3_)_3_), 2.82 (s, 3H, CH_3_), 7.41–7.46 (m, 1H, Ar), 7.49–7.55 (m, 4H, Ar), 7.73 (d, 2H, Ar, *J =* 8.4 Hz), 7.99 (d, 2H, Ar, *J =* 8.8 Hz). ^13^C NMR (CDCl_3_-d_1_) *δ* 15.11 (CH_3_), 27.56 (CH_3_), 39.95 (C), 118.86 (CH), 126.01 (C), 127.54 (C), 127.66 (CH), 128.47 (CH), 128.82 (CH), 129.05 (CH), 131.65 (CH), 142.83 (C), 154.74 (C), 162.65 (C), 166.04 (C), 176.92 (C). ESI-MS calcd. for C_22_H_22_N_2_O_4_, 378.42; found: *m/z* 379.16 [M + H]^+^. Anal. C_22_H_22_N_2_O_4_ (C, H, N).

##### 4-(3-Methyl-5-oxo-4-phenyl-2,5-dihydroisoxazole-2-carbonyl)phenyl pivalate (4l)

Yield* =* 41%; mp* =* 130–132 °C (EtOH). ^1^H NMR (CDCl_3_-d_1_) *δ* 1.37 (s, 9H, C(CH_3_)_3_), 2.80 (s, 3H, CH_3_), 7.22 (d, 2H, Ar, *J =* 8.8 Hz), 7.35–7.40 (m, 1H, Ar), 7.45–7.50 (m, 4H, Ar), 7.99 (d, 2H, Ar, *J =* 8.8 Hz). ^13^C NMR (CDCl_3_-d_1_) *δ* 15.07 (CH_3_), 27.07 (CH_3_), 39.26 (C), 108.25 (C), 121.70 (CH), 127.56 (C), 128.16 (CH), 128.53 (CH), 128.83 (CH), 129.04 (CH), 131.70 (CH), 154.55 (C), 155.05 (C), 162.54 (C), 165.80 (C), 176.34 (C). IR (*υ*)* =* 1678 cm^−1^ (CO amide), 1753 cm^−1^ (CO ester), 1768 cm^−1^ (CO ester). ESI-MS calcd. for C_22_H_21_NO_5_, 379.41; found: *m/z* 380.15 [M + H]^+^. Anal. C_22_H_21_NO_5_ (C, H, N).

#### General procedure for compounds (4m, 4u)

To a solution of intermediate **1b**^42^ or **1f**^47^ (0.32 mmol) in 2 ml of t-BuOH, 0.35 mmol of K_2_CO_3_ and 0.64 mmol of *m*-toluoyl chloride were added. The mixture was stirred at reflux for 3 h. The solvent was concentrated *in vacuo*, diluted with ice-cold water (10 ml), and extracted with DCM (3 × 15 ml). The organic phase was dried over sodium sulphate and the solvent was evaporated *in vacuo* to afford the final compounds **4m,u**, which were purified by column chromatography using cyclohexane/ethyl acetate in different ratio: 5:1 for **4m** and 1:1 for **4u** as eluent.

##### 2-(3-Methylbenzoyl)-3,4-diphenylisoxazol-5(2H)-one (4m)

Yield* =* 14%; mp* =* 160–163 °C (EtOH). ^1^H NMR (CDCl_3_-d_1_) *δ* 2.42 (s, 3H, m-*CH_3_*-Ph), 7.26–7.32 (m, 5H, Ar), 7.37–7.48 (m, 7H, Ar), 7.72–7.77 (m, 2H, Ar). ^13^C NMR (CDCl_3_-d_1_) *δ* 21.40 (CH_3_), 101.40 (C), 127.48 (CH), 128.31 (CH), 128.46 (CH), 128.53 (CH), 128.83 (CH), 130.74 (CH), 132.49 (C), 132.63 (C), 134.21 (C), 134.52 (CH), 138.50 (C), 142.65 (C), 157.66 (C), 165.80 (C). ESI-MS calcd. for C_23_H_17_NO_3_, 355.39; found: *m/z* 356.12 [M + H]^+^. Anal. C_23_H_17_NO_3_ (C, H, N).

##### N-(4-(2-(3-methylbenzoyl)-5-oxo-2,5-dihydroisoxazol-3-yl)phenyl)acetamide (4u)

Yield* =* 21%; oil. ^1^H NMR (CDCl_3_-d_1_) *δ* 2.20 (s, 3H, CH_3_CO), 2.45 (s, 3H, m-*CH_3_*-Ph), 6.50 (s, 1H, CH), 7.42 (t, 1H, Ar, *J =* 7.8 Hz), 7.50 (d, 1H, Ar, *J =* 7.6 Hz), 7.56 (exch br s, 1H, NH), 7.62 (d, 2H, Ar, *J =* 8.0 Hz), 7.76 (d, 2H, Ar, *J =* 8.4 Hz), 7.98–8.13 (m, 2H, Ar). ^13^C NMR (CDCl_3_-d_1_) *δ* 21.30 (CH_3_), 24.81 (CH_3_), 85.70 (C), 119.72 (CH), 127.45 (CH), 127.93 (CH), 128.88 (CH), 129.65 (C), 131.17 (CH), 134.11 (C), 135.75 (CH), 138.99 (C), 139.73 (C), 156.05 (C), 157.65 (C), 167.14 (C), 168.90 (C). ESI-MS calcd. for C_19_H_16_N_2_O_4_, 336.34; found: *m/z* 337.11 [M + H]^+^. Anal. C_19_H_16_N_2_O_4_ (C, H, N).

#### General procedure for compounds (5e, 5f)

To a cooled (0 °C) suspension of **5d**^48^ (0.68 mmol) in anhydrous CH_2_Cl_2_ (2 ml), Et_3_N (1.36 mmol) and 2.04 mmol of appropriate acyl chloride were added. The mixture was stirred at 0 °C for 2 h and then at room temperature for an additional 2 h. The solvent was evaporated, cold water was added and the mixture was neutralised with 0.5 N NaOH. The reaction mixture was extracted with CH_2_Cl_2_ (3 × 15 ml), the solvent was dried over sodium sulphate, evaporated *in vacuo*, and compounds **5e** and **5f** were purified by column chromatography using dichloromethane/methanol (9:1 for **5e**; 99:1 for **5f**) as eluents.

##### Ethyl 2-(4-acetamidophenyl)-3-oxobutanoate (5e)

Yield* =* 67%; mp* =* 112–113 °C (EtOH). ^1^H NMR (CDCl_3_-d_1_) showed a 3:1 mixture of aldo-enol tautomers: *δ* 1.24 (t, 3H, CH_2_*CH_3_*, *J* = 7.0 Hz), 1.61 (t, 1H, CH_2_*CH_3_*, *J* = 7.0 Hz), 1.81 (s, 1H, COCH_3_), 2.12 (s, 3H, COCH_3_), 2.15 (s, 4H, NHCOCH_3_), 4.16 (q, 2.7H, *CH_2_*CH_3_, *J* = 7.2 Hz), 4.65 (s,1H, CH), 7.06 (d, 0.7H, Ar, *J* = 8.0 Hz), 7.23 (d, 2H, Ar, *J* = 8.4 Hz), 7.48 (d, 2.7H, Ar, *J* = 8.0 Hz), 7.92 (exch br s, 1.3H, NH), 13.07 (exch br s, 0.3H, OH). ESI-MS calcd. for C_14_H_17_NO_4_, 263.29; found: *m/z* 264.12 [M + H]^+^. Anal. C_14_H_17_NO_4_ (C, H, N).

##### Ethyl 2-(4-(ciclopropanecarboxamido)phenyl)-3-oxobutanoate (5f)

Yield* =* 78%; oil. ^1^H NMR (CDCl_3_-d_1_) showed the only aldo tautomer: *δ* 0.72–0.77 (m, 2H, CH_2_ cC_3_H_5_), 0.80–0.85 (m, 2H, CH_2_ cC_3_H_5_), 1.20 (t, 3H, CH_2_*CH_3_*, *J* = 7.2 Hz), 1.39–1.46 (m, 1H, CH cC_3_H_5_), 2.35 (s, 3H, COCH_3_), 4.17 (q, 2H, *CH_2_*CH_3_, *J* = 7.2 Hz), 4.63 (s, 1H, CH), 7.19 (d, 2H, Ar, *J* = 8.4 Hz), 7.46 (d, 2H, Ar, *J* = 7.6 Hz), 7.52 (exch br s, 1H, NH). ESI-MS calcd. for C_16_H_19_NO_4_, 289.33; found: *m/z* 290.13 [M + H]^+^. Anal. C_16_H_19_NO_4_ (C, H, N).

#### General procedure for compounds (6a–e)

A 3.00 mmol of appropriate intermediate **5a–c**^48–50^ and **5e**,**f** was dissolved in 1.3 ml of water and heated at 80 °C. To this solution, 3.3 mmol of hydroxylamine hydrochloride in 6.5 ml of methanol was added. The mixture was stirred at reflux for 5 h. After evaporation of the solvent, the residue was mixed with ice-cold water (20 ml). Compounds **6a,c** were recovered by extraction with ethyl acetate (3 × 15 ml), while compounds **6b,d,e** were recovered by vacuum filtration. The final compounds **6b,d** were purified by crystallisation with ethanol, while the compounds **6a,c,e** were purified by column chromatography using dichoromethane/methanol 9:1 as eluent.

##### 3-Methyl-4-(p-tolyl)isoxazol-5(2H)-one (6a)

Yield* =* 35%; oil. ^1^H NMR (CDCl_3_-d_1_) *δ* 2.17 (s, 3H, *CH_3_*Ph), 2.28 (s, 3H, CH_3_), 7.09 (d, 2H, Ar, *J* = 6.0 Hz), 7.28 (d, 2H, Ar, *J* = 6.0 Hz), 9.03 (exch br s, 1H, NH). ESI-MS calcd. for C_11_H_11_NO_2_, 189.21; found: *m/z* 190.08 [M + H]^+^. Anal. C_11_H_11_NO_2_ (C, H, N).

##### 4-(3-Methyl-5-oxo-2,5-dihydroisoxazol-4-yl)benzonitrile (6b)

Yield* =*  72%; mp* =* 114–116 °C (EtOH). ^1^H NMR (CDCl_3_-d_1_) *δ* 2.43 (s, 3H, CH_3_), 7.70 (s, 4H, Ar). IR (*υ*)* =* 2270 cm^−1^ (CN). ESI-MS calcd. for C_11_H_8_N_2_O_2_, 200.19; found: *m/z* 201.06 [M + H]^+^. Anal. C_11_H_8_N_2_O_2_ (C, H, N).

##### 3-Methyl-4-(4-nitrophenyl)isoxazol-5(2H)-one (6c)

Yield* =* 27%; mp* =* 208–209 °C (EtOH). ^1^H NMR (DMSO-d_6_) *δ* 2.19 (s, 3H, CH_3_), 7.83 (d, 2H, Ar, *J* = 9.2 Hz), 7.94 (d, 2H, Ar, *J* = 8.4 Hz). ESI-MS calcd. for C_10_H_8_N_2_O_4_, 220.18; found: *m/z* 221.05 [M + H]^+^. Anal. C_10_H_8_N_2_O_4_ (C, H, N).

##### N-(4-(3-methyl-5-oxo-2,5-dihydroisoxazol-4-yl)phenyl)acetamide (6d)

Yield* =* 75%; mp* =* 210–212 °C (EtOH). ^1^H NMR (DMSO-d_6_) *δ* 2.01 (s, 3H, NHCO*CH_3_*), 2.27 (s, 3H, CH_3_), 7.43 (d, 2H, Ar, *J* = 8.0 Hz); 7.56 (d, 2H, Ar, *J* = 8.0 Hz), 9.92 (exch br s, 1H, *NH*COCH_3_), 12.54 (exch br s, 1H, NH). ESI-MS calcd. for C_12_H_12_N_2_O_3_, 232.24; found: *m/z* 233.09 [M + H]^+^. Anal. C_12_H_12_N_2_O_3_ (C, H, N).

##### N-(4-(3-methyl-5-oxo-2,5-dihydroisoxazol-4-yl)phenyl)cyclopropanecarboxamide (6e)

Yield* =* 58%; mp* =* 208–210 °C (EtOH). ^1^H NMR (DMSO-d_6_) *δ* 0.70–0.75 (m, 4H, 2 × CH_2_ cC_3_H_5_), 1.70–1.75 (m,1H, CH), 2.12 (s, 3H, CH_3_), 7.40 (d, 2H, Ar, *J* = 8.4 Hz), 7.47 (d, 2H, Ar, *J* = 8.4 Hz), 9.97 (exch br s, 1H, *NH*CO cC_3_H_5_), 12.50 (exch br s, 1H, NH). ESI-MS calcd. for C_14_H_14_N_2_O_3_, 258.27; found: *m/z* 259.10 [M + H]^+^. Anal. C_14_H_14_N_2_O_3_ (C, H, N).

#### General procedure for compounds (7a–e, 8a,b,d,e)

Compounds **7a–e** and **8a,b,d,e** were obtained following the same procedure performed for compounds **4a–h, 4n–t** but starting from precursors **6a–e**. The solvent was concentrated *in vacuo* to obtain the final compounds which were purified by column chromatography using petroleum ether/ethyl acetate 10:1 for **7a**/**8a**, hexane/ethyl acetate (5:1 for **7b/8b**; 5:2 for **7c**), dichloromethane/methanol (98:2 for **7d/8d**; 99:1 for **7e/8e**) as eluents.

##### 3-Methyl-2-(3-methylbenzoyl)-4-(p-tolyl)isoxazol-5(2H)-one (7a)

Yield* =* 60%; oil. ^1^H NMR (CDCl_3_-d_1_) *δ* 2.39 (s, 3H, *p-CH_3_*Ph), 2.43 (s, 3H, *m-CH_3_*Ph), 2.79 (s, 3H, CH_3_), 7.27 (d, 2H, Ar, *J* = 8.4 Hz), 7.37–7.42 (m, 4H, Ar), 7.70 (d, 2H, Ar, *J* = 6.8 Hz). ^13 ^C NMR (CDCl_3_-d_1_) *δ* 15.05 (CH_3_), 21.33 (CH_3_), 108.19 (C), 124.63 (C), 127.03 (CH), 128.30 (CH), 128.89 (CH), 129.52 (CH), 130.24 (CH), 131.27 (C), 134.00 (CH), 138.28 (C), 138.48 (C), 154.09 (C), 163.82 (C), 166.07 (C). ESI-MS calcd. for C_19_H_17_NO_3_, 307.34; found: *m/z* 308.12 [M + H]^+^. Anal. C_19_H_17_NO_3_ (C, H, N).

##### 4-(3-Methyl-2-(3-methylbenzoyl)-5-oxo-2,5-dihydroisoxazol-4-yl)benzonitrile (7b)

Yield* =* 26%; mp* =* 108–110 °C (EtOH). ^1^H NMR (CDCl_3_-d_1_) *δ* 2.44 (s, 3H, *CH_3_*Ph), 2.84 (s, 3H, CH_3_), 7.38–7.48 (m, 2H, Ar), 7.65 (d, 2H, Ar, *J* = 8.4 Hz), 7.71 (d, 2H, Ar, *J* = 8.0 Hz), 7.76 (d, 2H, Ar, *J* = 8.4 Hz). ^13^C NMR (CDCl_3_-d_1_) *δ* 15.19 (CH_3_), 21.37 (CH_3_), 29.70 (C), 106.24 (C), 112.03 (C), 118.41 (C), 127.13 (CH), 128.43 (CH), 129.43 (CH), 130.34 (CH), 130.72 (C), 132.52 (CH), 132.74 (C), 134.45 (CH), 138.49 (C), 155.56 (C), 163.79 (C), 165.15 (C). ESI-MS calcd. for C_19_H_14_N_2_O_3_, 318.33; found: *m/z* 319.10 [M + H]^+^. Anal. C_19_H_14_N_2_O_3_ (C, H, N).

##### 3-Methyl-2-(3-methylbenzoyl)-4-(4-nitrophenyl)isoxazol-5(2H)-one (7c)

Yield* =* 6%; mp* =* 152–154 °C (EtOH). ^1^H NMR (CDCl_3_-d_1_) *δ* 2.45 (s, 3H, *CH_3_*Ph), 2.86 (s, 3H, CH_3_), 7.39–7.46 (m, 2H, Ar), 7.72 (d, 4H, Ar, *J* = 8.8 Hz), 8.32 (d, 2H, Ar, *J* = 8.8 Hz). ^13^C NMR (CDCl_3_-d_1_) *δ* 13.21 (CH_3_), 21.32 (CH_3_), 29.70 (C), 124.02 (CH), 127.16 (CH), 128.45 (CH), 129.56 (CH), 130.36 (CH), 130.65 (C), 134.51 (CH), 134.69 (C), 138.52 (C), 147.37 (C), 155.78 (C), 163.80 (C). ESI-MS calcd. for C_18_H_14_N_2_O_5_, 338.31; found: *m/z* 339.09 [M + H]^+^. Anal. C_18_H_14_N_2_O_5_ (C, H, N).

##### N-(4-(3-methyl-2-(3-methylbenzoyl)-5-oxo-2,5-dihydroisoxazol-4-yl)phenyl)acetamide (7d)

Yield* =* 37%; mp* =* 159–160 °C (EtOH). ^1^H NMR (CDCl_3_-d_1_) *δ* 2.14 (s, 3H, NHCO*CH_3_*), 2.41 (s, 3H, *CH_3_*Ph), 2.75 (s, 3H, CH_3_), 7.34–7.40 (m, 4H, Ar), 7.58 (d, 2H, Ar, *J* = 8.4 Hz), 7.67 (s, 2H, Ar), 7.97 (exch br s, 1H, NH). ^13^C NMR (CDCl_3_-d_1_) *δ* 15.06 (CH_3_), 21.35 (CH_3_), 24.53 (CH_3_), 120.11 (CH), 123.11 (C), 126.99 (CH), 128.33 (CH), 128.94 (CH), 129.61 (CH), 130.23 (CH), 131.11 (C), 131.27 (CH), 134.12 (CH), 138.29 (C), 154.40 (C), 163.79 (C), 166.26 (C), 168.87 (C). ESI-MS calcd. for C_20_H_18_N_2_O_4_, 350.37; found: *m/z* 351.13 [M + H]^+^. Anal. C_20_H_18_N_2_O_4_ (C, H, N).

##### N-(4-(3-methyl-2-(3-methylbenzoyl)-5-oxo-2,5-dihydroisoxazol-4-yl)phenyl)ciclopropanecarboxamide (7e)

Yield* =* 6%; mp* =* 120–123 °C (EtOH). ^1^H NMR (CDCl_3_-d_1_) *δ* 0.84–0.89 (m, 2H, CH_2_ cC_3_H_5_), 1.07–1.12 (m, 2H, CH_2_ cC_3_H_5_), 1.24 (s, 1H, CH), 2.43 (s, 3H, *CH_3_*Ph), 2.79 (s, 3H, CH_3_), 7.36–7.41 (m, 2H, Ar), 7.46 (d, 2H, Ar, *J* = 8.4 Hz), 7.61 (d, 2H, Ar, *J* = 8.4 Hz), 7.70 (d, 2H, Ar, *J* = 7.2 Hz). ^13^C NMR (CDCl_3_-d_1_) *δ* 8.24 (CH_2_), 15.13 (CH_3_), 15.88 (CH), 21.41 (CH_3_), 107.20 (C), 119.79 (CH), 127.03 (CH), 128.33 (CH), 129.68 (CH), 130.25 (CH), 130.30 (C), 134.09 (CH), 134.10 (C), 137.70 (C), 138.33 (C), 139.50 (C), 157.61 (C), 163.83 (C), 180.70 (C). ESI-MS calcd. for C_22_H_20_N_2_O_4_, 376.41; found: *m/z* 377.15 [M + H]^+^. Anal. C_22_H_20_N_2_O_4_ (C, H, N).

##### 3-Methyl-4-(p-tolyl)isoxazol-5-yl 3-methylbenzoate (8a)

Yield* =* 12%; oil. ^1^H NMR (CDCl_3_-d_1_) *δ* 2.33 (s, 3H, *p-CH_3_*Ph), 2.38 (s, 3H, CH_3_), 2.41 (s, 3H, *m-CH_3_*Ph), 7.18 (d, 2H, Ar, *J* = 8.0 Hz), 7.24 (d, 2H, Ar, *J* = 8.4 Hz), 7.38 (t, 1H, Ar, *J* = 8.0 Hz), 7.47 (d, 1H, Ar, *J* = 7.2 Hz), 7.93 (s, 2H, Ar). ^13 ^C NMR (CDCl_3_-d_1_) *δ* 12.21 (CH_3_), 21.23 (CH_3_), 100.50 (C), 127.97 (CH), 128.10 (CH), 128.75 (CH), 129.63 (CH), 130.10 (C), 131.26 (CH), 133.30 (C), 135.57 (CH), 138.84 (C), 154.20 (C), 158.90 (C), 165.20 (C). ESI-MS calcd. for C_19_H_17_NO_3_, 307.34; found: *m/z* 308.12 [M + H]^+^. Anal. C_19_H_17_NO_3_ (C, H, N).

##### 4-(4-Cyanophenyl)-3-methylisoxazol-5-yl 3-methylbenzoate (8b)

Yield* =* 20%; mp* =* 98–100 °C (EtOH). ^1^H NMR (CDCl_3_-d_1_) *δ* 2.41 (s, 3H, CH_3_), 2.43 (s, 3H, *CH_3_*Ph), 7.43 (t, 1H, Ar, *J* = 8.0 Hz), 7.47 (d, 2H, Ar, *J* = 8.4 Hz), 7.51 (d, 1H, Ar, *J* = 7.6 Hz), 7.67 (d, 2H, Ar, *J* = 8.4 Hz), 7.92 (s, 2H, Ar). ^13 ^C NMR (CDCl_3_-d_1_) *δ* 12.31 (CH_3_), 21.25 (CH_3_), 29.70 (C), 111.68 (C), 118.37 (C), 126.33 (C), 128.02 (CH), 128.61 (CH), 128.95 (CH), 131.30 (CH), 132.75 (CH), 133.27 (C), 136.04 (CH), 139.11 (C), 160.61 (C), 161.90 (C). ESI-MS calcd. for C_19_H_14_N_2_O_3_, 318.33; found: *m/z* 319.10 [M + H]^+^. Anal. C_19_H_14_N_2_O_3_ (C, H, N).

##### 4-(4-Acetamidophenyl)-3-methylisoxazol-5-yl3-methylbenzoate (8d)

Yield* =* 10%; mp* =* 57–60 °C (EtOH). ^1^H NMR (CDCl_3_-d_1_) *δ* 2.15 (s, 3H, NHCO*CH_3_*), 2.36 (s, 3H, CH_3_), 2.41 (s, 3H, *CH_3_*Ph), 7.29 (d, 3H, Ar, *J* = 8.4 Hz), 7.38 (t, 1H, Ar, *J* = 8.0 Hz), 7.46 (exch br s, 1H, NH), 7.51 (d, 2H, Ar, *J* = 8.4 Hz), 7.91 (s, 2H, Ar). ^13^C NMR (CDCl_3_-d_1_) *δ* 12.23 (CH_3_), 21.24 (CH_3_), 24.59 (CH_3_), 29.69 (C), 120.11 (CH), 123.90 (C), 126.71 (C), 127.97 (CH), 128.83 (CH), 129.67 (CH), 131.26 (CH), 135.70 (CH), 137.61 (C), 138.91 (C), 161.21 (C), 162.31 (C), 168.39 (C). ESI-MS calcd. for C_20_H_18_N_2_O_4_, 350.37; found: *m/z* 351.13 [M + H]^+^. Anal. C_20_H_18_N_2_O_4_ (C, H, N).

##### 4-(4-(Ciclopropanecarboxamido)phenyl)-3-methylisoxazol-5-yl3-me-thylbenzoate (8e)

Yield* =* 8%; mp* =* 80–82 °C (EtOH). ^1^H NMR (CDCl_3_-d_1_) *δ* 0.80–0.90 (m, 4H, 2 × CH_2_ cC_3_H_5_), 1.35–1.40 (m, 1H, CH), 2.37 (s, 3H, CH_3_), 2.41 (s, 3H, *CH_3_*Ph), 7.28 (d, 2H, Ar, *J* = 8.0 Hz), 7.38 (t, 1H, Ar, *J* = 7.6 Hz), 7.40 (exch br s,1H, NH), 7.47 (d, 1H, Ar, *J* = 7.6 Hz), 7.52 (d, 2H, Ar, *J* = 7.2 Hz), 7.92 (s, 2H, Ar). ^13^C NMR (CDCl_3_-d_1_) *δ* 8.15 (CH_2_), 12.24 (CH_3_), 14.90 (CH), 21.25 (CH_3_), 100.52 (C), 119.91 (CH), 127.97 (CH), 128.79 (CH), 128.85 (CH), 131.25 (CH), 132.05 (C), 135.68 (CH), 138.70 (C), 138.89 (C), 154.20 (C), 158.90 (C), 165.20 (C), 180.70 (C). ESI-MS calcd. for C_22_H_20_N_2_O_4_, 376.41; found: *m/z* 377.15 [M + H]^+^. Anal. C_22_H_20_N_2_O_4_ (C, H, N).

#### General procedure for compounds (10a–c)

To suspension of the substrate **9**^51^ (0.37 mmol) in *tert*-Butanol (3 ml), K_2_CO_3_ (0.41 mmol), and 0.74 mmol of the appropriate acyl chloride were added. The mixture was stirred at reflux for 3 h. After evaporation of the solvent, the residue was mixed with ice-cold water (20 ml) and extracted with ethyl acetate (3 × 15 ml). The organic phase was dried over sodium sulphate, and the solvent was evaporated *in vacuo* to afford the final compounds 1**0a–c**, which were purified by column chromatography using cyclohexane/ethyl acetate (5:1) as eluent.

##### 1-Propionylbenzo[c]isoxazol-3(1H)-one (10a)

Yield* =* 7%; oil. ^1^H NMR (CDCl_3_-d_1_) *δ* 1.28 (t, 3H, CH_2_*CH_3_*, *J* = 7.4 Hz), 2.83 (q, 2H, *CH_2_*CH_3_, *J =* 7.2 Hz), 7.38 (t 1H, Ar, *J =* 7.6 Hz), 7.78 (t 1H, Ar, *J =* 7.6 Hz), 7.89 (d, 1H, Ar, *J =* 8.0 Hz), 8.11 (d, 1H, Ar, *J =* 8.4 Hz). ^13^C NMR (CDCl_3_-d_1_) *δ* 9.72 (CH_3_), 20.70 (CH_2_), 120.35 (CH), 122.50 (C), 124.09 (CH), 130.31 (CH), 133.90 (CH), 142.44 (C), 166.02 (C), 172.05 (C). ESI-MS calcd. for C_10_H_9_NO_3_, 191.18; found: *m/z* 192.06 [M + H]^+^. Anal. C_10_H_9_NO_3_ (C, H, N).

##### 1-Pentanoylbenzo[c]isoxazol-3(1H)-one (10b)

Yield* =* 12%; oil. ^1^H NMR (CDCl_3_-d_1_) *δ* 0.87 (t, 3H, *CH_3_*CH_2_CH_2_CH_2_CO, *J =* 6.8 Hz), 1.53–1.58 (m, 2H, CH_3_*CH_2_*CH_2_CH_2_CO), 1.98–2.03 (m, 2H, CH_3_CH_2_*CH_2_*CH_2_CO), 3.63 (t, 2H, CH_3_CH_2_CH_2_*CH_2_*CO, *J =* 6.8 Hz), 7.22 (d, 1H, Ar, *J =* 8.4 Hz), 7.31 (t, 1H, Ar, *J =* 7.4 Hz), 7.68 (t, 1H, Ar, *J =* 7.6 Hz), 7.86 (d, 1H, Ar, *J =* 7.6 Hz). ^13^C NMR (CDCl_3_-d_1_) *δ* 13.10 (CH_3_), 22.15 (CH_2_), 27.65 (CH_2_), 28.21 (CH_2_), 120.31 (CH), 122.49 (C), 124.00 (CH), 130.33 (CH), 133.90 (CH), 142.41 (C), 165.31 (C), 172.22 (C). ESI-MS calcd. for C_12_H_13_NO_3_, 219.24; found: *m/z* 220.09 [M + H]^+^. Anal. C_12_H_13_NO_3_ (C, H, N).

##### 1-(3-Methylbenzoyl)benzo[c]isoxazol-3(1H)-one (10c)

Yield* =* 53%; mp* =* 116–119 °C (EtOH). ^1^H NMR (CDCl_3_-d_1_) *δ* 2.44 (s, 3H, CH_3_), 7.39–7.45 (m, 3H, Ar), 7.75–7.80 (m, 2H, Ar), 7.83 (t 1H, Ar, *J =* 8.4 Hz), 7.92 (d, 1H, Ar, *J =* 8.0 Hz), 8.22 (d, 1H, Ar, *J =* 8.4 Hz). ^13^C NMR (CDCl_3_-d_1_) *δ* 21.40 (CH_3_), 115.83 (CH), 117.51 (C), 125.60 (CH), 126.03 (CH), 126.87 (CH), 128.34 (CH), 130.12 (CH), 133.40 (C), 133.81 (CH), 136.54 (CH), 137.80 (C), 151.60 (C), 158.51 (C), 172.03 (C). ESI-MS calcd. for C_15_H_11_NO_3_, 253.25; found: *m/z* 254.08 [M + H]^+^. Anal. C_15_H_11_NO_3_ (C, H, N).

### HNE inhibition assay

Compounds were dissolved in 100% DMSO at 5 mM stock concentrations. The final concentration of DMSO in the reactions was 1%, and this level of DMSO had no effect on enzyme activity. The HNE inhibition assay was performed in black flat-bottom 96-well microtiter plates. Briefly, a buffer solution containing 200 mM Tris–HCl, pH 7.5, 0.01% bovine serum albumin, and 0.05% Tween-20 and 20 mU/mL of HNE (Calbiochem) was added to wells containing different concentrations of each compound. The reaction was initiated by addition of 25 µM elastase substrate (N-methylsuccinyl-Ala-Ala-Pro-Val-7-amino-4-methylcoumarin, Calbiochem) in a final reaction volume of 100 µl/well. Kinetic measurements were obtained every 30 s for 10 min at 25 °C using a Fluoroskan Ascent FL fluorescence microplate reader (Thermo Electron, MA) with excitation and emission wavelengths set at 355 and 460 nm, respectively. For all compounds tested, the concentration of inhibitor that caused 50% inhibition of the enzymatic reaction (IC_50_) was calculated by plotting % inhibition versus logarithm of inhibitor concentration (at least six points). The data are presented as the mean values of at least three independent experiments with relative standard deviations of <15%.

### Analysis of compound stability

Spontaneous hydrolysis of selected derivatives was evaluated at 25 °C in 0.05 M phosphate buffer, pH 7.3. Kinetics of hydrolysis were monitored by measuring changes in the absorbance spectra over time using a SpectraMax Plus microplate spectrophotometer (Molecular Devices, Sunnyvale, CA). Absorbance (*A*_t_) at the characteristic absorption maxima of each compound was measured at the indicated times until no further absorbance decreases occurred (*A*_∞_)^52^. Using these measurements, we created semilogarithmic plots of log(*A*_t_–*A*_∞_) versus time, and *k*′ values were determined from the slopes of these plots. Half-conversion times were calculated using *t*_1/2_ = 0.693/*k*′, as described previously^36^^,^^37^.

### Molecular modelling procedures

The programs used for the energy minimisation, MD, and docking were the simulation protocols Minimisation, Standard Dynamics Cascade, Analyse Trajectory, and CDocker implemented in Accelrys Discovery Studio 2.1^53^. The Force Field used for all simulations was CHARMm^54^.

The following parameters were used for MD simulations, both in vacuum and in implicit solvent (the latter was simulated by using distance dependent dielectric constant set to 4r): time step* =* 1 fs, equilibration time* =* 100 ps, production time* =* 1000 ps (5000 ps for the inhibitor-HNE assembly), *T* = 300 and 600 K. Ten snapshot conformations with evenly spaced intervals were extracted from each MD trajectory and subsequently minimised (using the steepest descent and conjugate gradient algorithms) in order to obtain the starting geometries for the subsequent quantum chemical calculations (QC) and MD simulations with the receptor.

gaussian09 (rev. C01)^55^ was used for quantum chemical calculations (QC) on **7d** and **8d** by using the B3LYP^56^^,^^57^ and B97D^58^ functionals. The basis set was 6-31 + G(d,p)^59^, and the Berny algorithm was used^60^. Reliability of the stationary points was assessed by evaluation of the vibrational frequencies. For each inhibitor, different conformational isomers, chosen from amongst the low lying energy conformers (as found in MD simulations and subsequent geometry optimisation) were considered.

Molecular plots were produced by the program Discovery Studio Visualiser (v 4.5)^61^.

## Results and discussion

### Chemistry

**Scheme 1.**
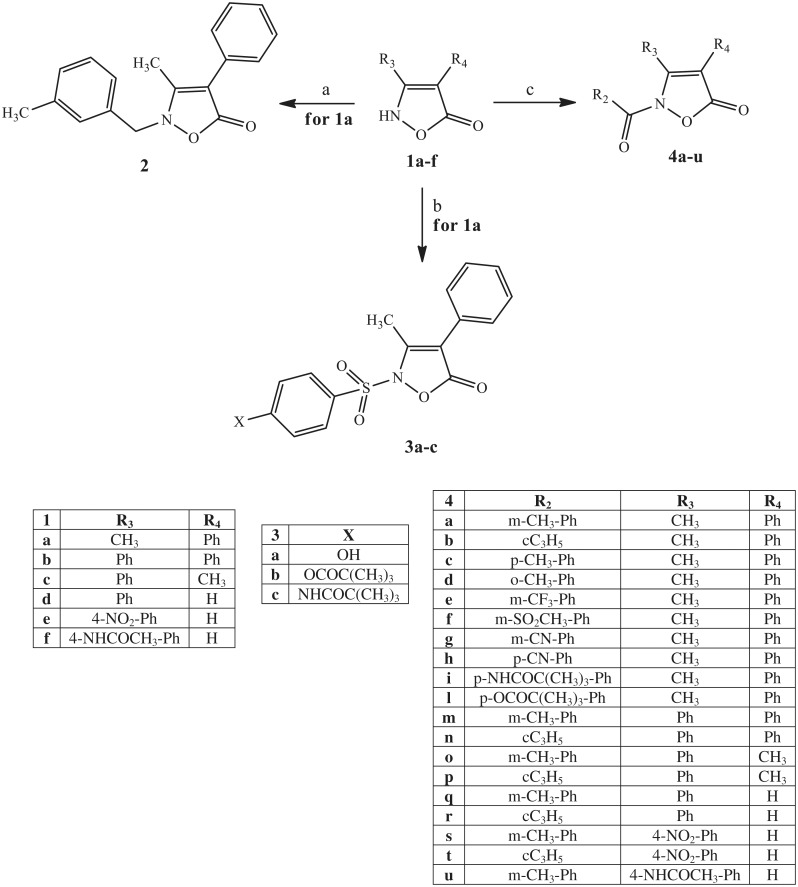
Reagents and conditions: (a) 3-methylbenzyl chloride, K_2_CO_3_, anhydrous CH_3_CN, 80 °C, 2 h; (b) 4-X-Ph-SO_2_Cl, anhydrous pyridine, r.t., 4 h; (c) for **4a–l, n–t:** R_2_-COCl, NaH, anhydrous THF, r.t., 24 h; for **4m,u**: m-toluoyl chloride, K_2_CO_3_, t-BuOH, 80 °C, 3 h.

**Scheme 2.**
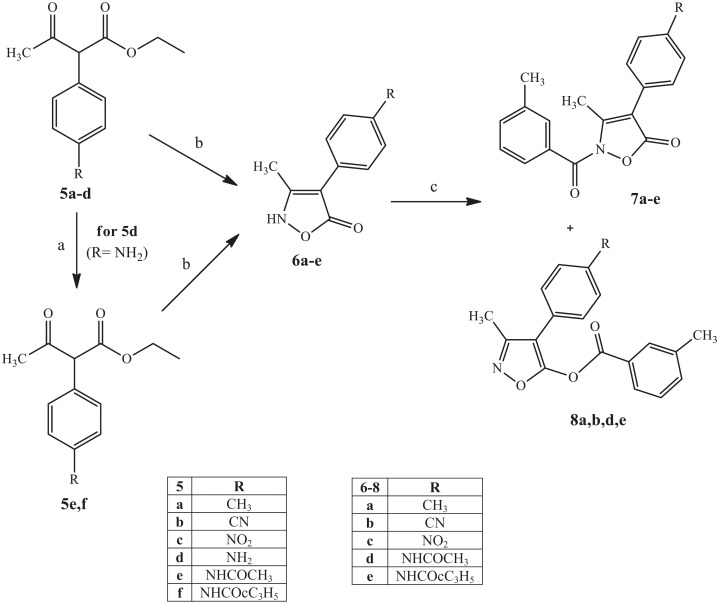
Reagents and conditions: (a) R-COCl, Et_3_N, anhydrous CH_2_Cl_2_, 0 °C, 2 h, then r.t., 2 h; (b) NH_2_OH.HCl, H_2_O/MeOH 1:1, reflux, 5 h; (c) m-toluoyl chloride, NaH, anhydrous THF, r.t., 24 h.

**Scheme 3.**
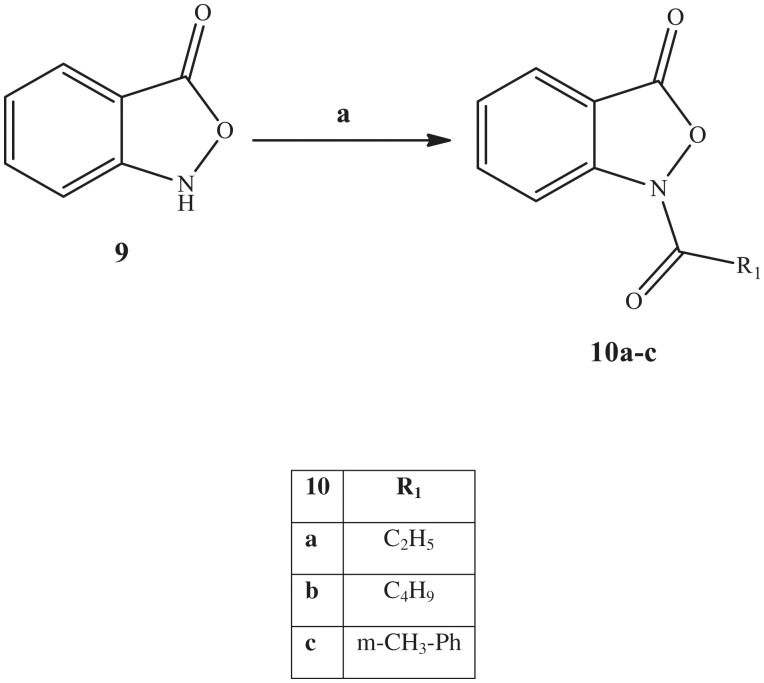
Reagents and conditions: (a) R_1_-COCl, K_2_CO_3_, t-BuOH, 80 °C, 3 h.

It is well-known that the isoxazolone nucleus exhibits three tautomers^43^^,^^62^^,^^63^, as illustrated below. The NH form seems to be the most representative, especially in polar solvents^62^^,^^64–66^. However, it is possible to find examples of alkylation and acylation products in the literature, as shown by the NH and the OH forms^67^. Taking into account this information, we performed the synthesis of our final compounds, as shown in Schemes 1–3, and the structures were confirmed on the basis of analytical and spectral data.

Scheme 1 depicts the synthetic pathway followed to obtain the final 2-N-substituted isoxazolones of type **2**, **3**, and **4**, which have different groups at positions 3 and 4. The previously described key intermediate isoxazol-5-(2H)-ones of type **1** were treated under various conditions to obtain the final compounds **2**, **3**, and **4**. The alkylation of **1a**^41^ with 3-methylbenzyl chloride and K_2_CO_3_ in anhydrous acetonitrile at reflux resulted in compound **2**. On the other hand, treatment of compounds **1a–f** (**1a**^41^, **1b**^42^, **1c,d**^43^, **1e**^44^, and **1f**^47^) with the appropriate acyl/aroyl chloride and NaH in anhydrous THF at room temperature (compounds **4a–l**,**n–t**) or with m-toluyl chloride, and K_2_CO_3_ in t-ButOH at 80 °C (compounds **4m,u**) resulted in the corresponding 2-NCO derivatives of type **4**. Likewise, we synthesised the sulfonamide derivatives **3a–c** by treatment of intermediate **1a**^41^ with the appropriate commercially available phenyl sulfonyl chloride in pyridine at room temperature. All of these reactions led to a single derivative originating from the NH form of the isoxazolone nucleus, in agreement with previous data reported in literature.

Scheme 2 shows the synthetic procedures used to obtain the final compounds of type **7** and **8**, which have a 4-substituted phenyl at position 4. β-Ketoesters **5a–d** (**5a**^49^, **5b,d**^48^, and **5c**^50^) were synthesised as described previously^48–50^, while compounds **5e,f** were obtained by acylation of **5d**^48^ with the appropriate acyl chloride and Et_3_N in dichloromethane. These compounds served as the starting material for synthesis of the key intermediates of type **6** with an isoxazolone nucleus. Cyclisation of **5a–c,e,f** with hydroxylamine hydrochloride in a mixture MeOH/H_2_O 1:1 at reflux resulted in the intermediates **6a–e** which, in turn, were treated with m-toluoyl chloride under the same conditions reported in Scheme 1. Unexpectedly this last step resulted the pair of isomers of type **7** and **8** (NCO/OCO ratio 3:1), with the only exception being the nitro derivative **7c**, which was obtained only in the N-CO form.

Assignment of the isomer structures was first performed using the ^1^H NMR chemical shift value of the methyl at position 3. Compounds **4a–l** (Scheme 1) had shifts of 2.8 ppm, which is characteristic of NCO derivatives. Likewise, we attributed the NCO-structure to compounds with a 2.8 ppm shift for 3-CH_3_ (**7a–e**) and the OCO-structure to compounds with a 2.4 ppm shift (**8a,b,d,e**) (see Supporting Information). In order to verify our findings, we performed additional techniques, such as IR spectroscopy and 2 D NMR (^1^H–^13^C HSQC, ^1^H–^13^C HMBC, and ^1^H–^1^H NOESY).

In Scheme 3, we show the synthetic pathways used to obtain the benzoisoxazolone derivatives **10a–c**, which are an elaboration of the previous isoxazolone scaffold. The acylation of benzoisoxazolone intermediate **9**^51^ with the appropriate acyl chloride and potassium carbonate in t-BuOH led to final compounds **10a–c**.

### Biological evaluation and structure–activity relationship (SAR) analysis

All compounds were evaluated for their ability to inhibit HNE in comparison with Sivelestat, a reference HNE inhibitor, and the results presented in Tables 1–3. Previously^40^, we examined the possibility of a two point of attack for Ser195, 2-NCO, and 5-CO, and docking studies confirmed that the endocyclic C = O at position 5 was involved in the catalysis process, whereas the amidic C = O group was important for anchoring to the sub-pocket of the binding site. We could expect a similar trend in this new series of isoxazolones.

**Table 1.** HNE inhibitory activity of isoxazolone derivatives **2**, **3a–c**, and **4a–u**.


Comp.	R_2_	R_3_	R_4_	IC_50_ (µM)^a^
**2**	**–**	**–**	**–**	NA
**3a**	p-OH-Ph	**–**	**–**	NA.
**3b**	p-OCOC(CH_3_)_3_-Ph	**–**	**–**	0.059 ± 0.02
**3c**	p-NHCOC(CH_3_)_3_-Ph	**–**	**–**	NA
**4a**	m-CH_3_-Ph	CH_3_	Ph	0.077 ± 0.027
**4b**	cC_3_H_5_	CH_3_	Ph	0.059 ± 0.018
**4c**	p-CH_3_-Ph	CH_3_	Ph	0.23 ± 0.034
**4d**	o-CH_3_-Ph	CH_3_	Ph	0.35 ± 0.036
**4e**	m-CF_3_-Ph	CH_3_	Ph	0.20 ± 0.027
**4f**	m-CH_3_SO_2_-Ph	CH_3_	Ph	29.6 ± 7.2
**4g**	m-CN-Ph	CH_3_	Ph	6.3 ± 1.4
**4h**	p-CN-Ph	CH_3_	Ph	11.4 ± 1.9
**4i**	p-NHCOC(CH_3_)_3_-Ph	CH_3_	Ph	8.5 ± 2.1
**4l**	p-OCOC(CH_3_)_3_-Ph	CH_3_	Ph	0.48 ± 0.13
**4m**	m-CH_3_-Ph	Ph	Ph	10.1 ± 1.3
**4n**	cC_3_H_5_	Ph	Ph	17.2 ± 2.3
**4o**	m-CH_3_-Ph	Ph	CH_3_	13.6 ± 2.4
**4p**	cC_3_H_5_	Ph	CH_3_	1.1 ± 0.14
**4q**	m-CH_3_-Ph	Ph	H	48.7 ± 3.3
**4r**	cC_3_H_5_	Ph	H	12.7 ± 2.7
**4s**	m-CH_3_-Ph	4-NO_2_-Ph	H	N.A.
**4t**	cC_3_H_5_	4-NO_2_-Ph	H	N.A.
**4u**	m-CH_3_-Ph	4-NHCOCH_3_-Ph	H	28.6 ± 3.3
**Sivelestat**				0.044 ± 0.011

a IC_50_ values are presented as the mean ± SD of three independent experiments.

NA: no inhibitory activity was found at the highest concentration of compound tested (50 µM).

**Table 2.** HNE inhibitory activity of isoxazolone derivatives **7a–e** and **8a,b,d,e**.


Comp.	R	IC_50_ (µM)^a^
**7a**	CH_3_	0.02 ± 0.009
**8a**	CH_3_	10.2 ± 1.4
**7b**	CN	0.07 ± 0.02
**8b**	CN	0.42 ± 0.13
**7c**	NO_2_	0.21 ± 0.04
**7d**	NHCOCH_3_	0.05 ± 0.02
**8d**	NHCOCH_3_	0.54 ± 0.11
**7e**	NHCOcC_3_H_5_	0.05 ± 0.02
**8e**	NHCOcC_3_H_5_	0.62 ± 0.21
**Sivelestat**		0.044 ± 0.011

a IC_50_ values are presented as the mean ± SD of three independent experiments.

**Table 3.** HNE inhibitory activity of benzoisoxazolone derivatives **10a–c**.


Comp.	R_1_	IC_50_ (µM)^a^
**10a**	C_2_H_5_	25.1 ± 3.6
**10b**	C_4_H_9_	NA
**10c**	m-CH_3_-Ph	0.638 ± 0.121
**Sivelestat**		0.044 ± 0.011

a IC_50_ values are presented as the mean ± SD of three independent experiments.

NA: no inhibitory activity was found at the highest concentration of compound tested (50 µM).

Beginning analysis of the data with the 3-methyl-4-phenylisoxazol-5(2H)-one derivatives (compounds **4a–l**), the results reported in Table 1 suggest that, similar to our previous series^40^, the best substituents at position N-2 are a m-methylbenzoyl or a cyclopropanecarbonyl fragment, which resulted in compounds **4a** and **4b** that were active in the nanomolar range (IC_50_* =* 77 and 59 nM, respectively). Displacement of the methyl group from the meta to the para (**4c**) or ortho (**4d**) positions on the phenyl led to compounds with activity one order of magnitude lower than **4a**, which is different than our previous series^40^, where moving the methyl to the para position had no effect on HNE inhibitory activity. Substitution of m-methyl with other groups, such as trifluoromethyl (**4e**), cyano (**4g,h**), or methylsulfonyl (**4f**), which are found in other potent HNE inhibitors, was not favourable for activity, and only compound **4e** exhibited activity in the submicromolar range (IC_50_* =* 200 nM).

To evaluate the importance of the 2-CO amidic group in this series, we synthesised the alkyl derivative **2**, which was completely inactive, suggesting that the carbonyl group is important for HNE inhibitory activity and also probably involved in catalysis. The insertion of the 3-methyl-4-phenylisoxazol-5(2H)-one scaffold of the sulfonamide fragment of the drug Sivelestat at position 2 led to compound **3b**, which exhibited comparable activity to Sivelestat (IC_50_ = 59 nM, Table 1). In contrast, its pivalamide derivative (**3c**) was completely inactive, suggesting that the possible point of attack of Ser195 is the CO of the pivalate function, which is also present in Sivelestat. These data were also confirmed by the inactivity of the hydrolysed derivative **3a**. Moreover, we synthesised compounds **4l** and **4i** by substituting the SO_2_ group at position 2 (**3b** and **3c**, respectively) with an amidic function. The amide **4l** had activity (IC_50_ = 0.48 µM) that was one order of magnitude higher than the corresponding sulfonamide **3b**, while the amide **4i** had a higher potency (IC_50_ = 8.5 µM) than its corresponding sulfonamide **3c**, which was completely inactive. These results suggest that the SO_2_ group at position 2 of **3b** could also be important for anchoring the ligand to the sub-pocket of the binding site.

Keeping m-methylbenzoyl and ciclopropanecarbonyl at position N-2, we modified positions 3 and 4 of the isoxazolone (Table 1). Generally, we observed a collapse of activity. In particular, the 3,4-diphenylisoxazol-5(2H)-one derivatives (compounds **4m** and **4n**), the 4-methyl-3-phenylisoxazol-5(2H)-one derivatives (compounds **4o** and **4p**), and the 3-phenylisoxazol-5(2H)-one derivatives (compounds **4q**,**r** and **4u**) all had decreased activity (IC_50_ = 10–50 µM). The insertion in the para position of the phenyl ring at position 3 of compounds **4q,r** with substituents that in the previous series gave good results (such as a nitro group or acetamide function) led to completely inactive (compounds **4s,t**). Based on the results in Table 1, we can conclude that position 4 can bear bulky groups, such as an aromatic ring, when there is a methyl group in position 3, probably to fit constraints of the lipophilic pocket. Moving the phenyl ring to position 3 is not tolerated, either when there is a methyl or a hydrogen present in position 4.

In Table 2, we report the activity of the pair of isomers of type **7** and **8**, the amidic and ester derivatives, respectively, which were obtained by introducing a substituent to the para position of the phenyl group at position 4. All N-benzoyl derivatives (**7a–e**), with the exception of the nitro compound **7c**, exhibited good HNE inhibitory activity in the nanomolar range (IC_50_* =* 20–70 nM). In addition, the activity seems to be independent of the electrophilic properties of the substituents (CH_3_, NO_2_, CN, NHCOR). The best compound of this series was the p-methyl derivative **7a**, which had an IC_50_ of 20 nM. The O-benzoyl derivatives **8b,d,e**, which were derived from tautomerism of the isoxazolone scaffold, surprisingly exhibited activity, although at one order of magnitude lower than the corresponding N-benzoyl type **7** derivatives (Table 2). Only the ester isomer of the potent **7a** had a significant loss in activity (**8a**, IC_50_ = 10 µM). The unexpected data related to esters **8a,b,d,e** could indicate a different interaction with the target, since the CO endocyclic implicated in the catalysis is missing, and the only carbonyl group presented in the molecule is an ester function at position 5. Further docking studies and kinetic experiments could help us to understand the interaction of these compounds with the HNE catalytic site and their mechanism of action.

Table 3 shows the HNE inhibitory activity of benzoisoxazolone derivatives **10a–c**, which are elaborations of the isoxazolone scaffold. In this case, only the m-methylbenzoyl derivative **10c** had reasonable activity, with an IC_50_ of 638 nM, while the other compounds were less active (**10a**) or inactive (**10b**).

### Molecular modelling

Several attempts were made using different solvents in order to obtain crystals of **7d** and **8d**. However, it was not possible to obtain crystals suitable for X-ray diffraction (probably in part due to their quite low melting points). As a consequence, the starting 3D geometry of these molecules was obtained by using as building blocks the solid state structures of 3,5-dicyano-4-(4-methoxyphenyl) isoxazole (QAQPON refcode)^68^ found in the Cambridge Structural Database (CSD; v 5.37)^69^ and that of the isoxazolone derivative **2j** (3-ethyl-2–(3-methylbenzoyl)isoxazol-5(2H)-one)^40^. The 3D arrangement of **7d** and **8d** was then roughly improved by an energy minimisation procedure, followed by molecular dynamics simulations (details in the Supplementary Material).

**Figure 2.**
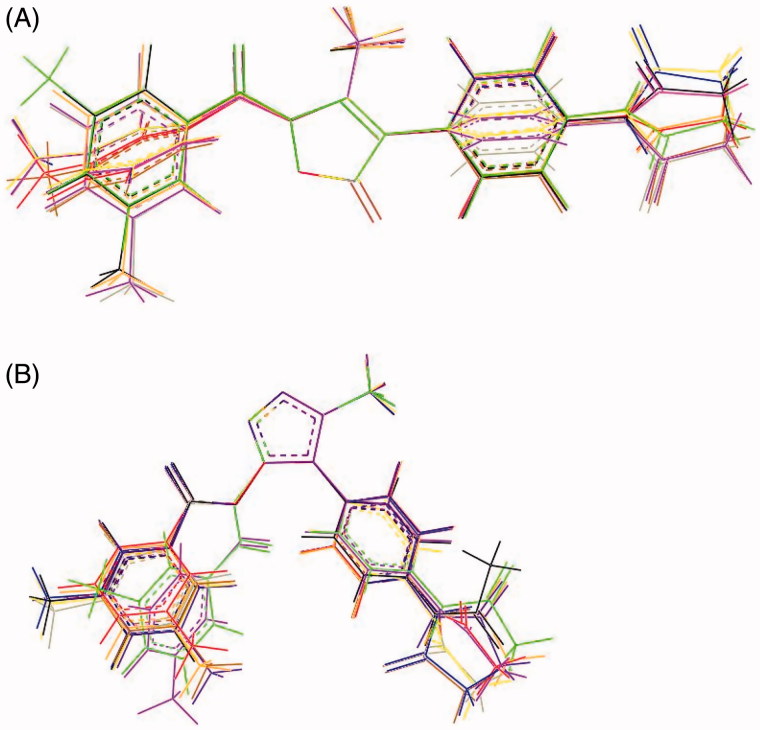
Panel (A): Superimposition of the minimised conformations of **7d** extracted from the MD trajectory (*T* = 600 K, *ε* = 4*r*). Panel (B): Superimposition of the minimised conformations of **8d** extracted from the MD trajectory (*T* = 600 K, *ε* = 4*r*).

Preliminary molecular dynamics (MD) simulations at 300 and 600 K were performed on **7d** and **8d** to evaluate their flexibility, accessible conformational space, and preferred 3D arrangements (low-energy conformations). As expected, the overall shape of both ligands did not change significantly on changing the simulation medium, as roughly determined by comparison of the dihedral angles, which define the overall shape of the molecules (see Figure S1, Supplementary Material). For **7d**, MD trajectories (300 and 600 K) showed that τ1 and τ2 adopted a trans conformation that is maintained throughout the simulations, while τ3–τ5 access different conformations (Figures S2 and S3, Supplementary Material). Overall, the molecule adopted an elongated cylindrical shape (Figure S4, Supplementary Material). In particular, the conformational behaviour of dihedrals τ1 and τ4 seems to be quite important (vide infra), given that the endocyclic C = O at position 5 appears to be involved in the catalytic process, while the closest C = O group could play an important role in anchoring to the sub-pocket of the binding site^40^. The superimposition (Figure 2, panel A) of the minimised conformers of **7d** extracted from the MD trajectory (*T* = 600 K, *ε* = 4*r*), which are comprised within about 2 kcal mol^−1^, exemplifies the conformational space accessible to the molecule through rotations about the τ3–τ5 dihedrals.

Compound **8d** appears to be a little bit more flexible compared to **7d**, as suggested by the larger variability of the distance separating the centroids of the phenyl rings. While the τ1, τ2, and τ3 dihedrals were almost frozen irrespective of the simulation temperature and medium [they adopted cis, trans (with only few exceptions), and cis (with only few exceptions) conformations, respectively], increasing the temperature from 300 to 600 K made the τ5 and τ6 dihedrals freely rotate (Figures S5 and S6, Supplementary Material). Dihedral τ4 showed a preference for a gauche arrangement. Overall, **8d** is V-shaped: in most cases both of the phenyl rings were rotated with respect to the mean plane defined by the heterocyclic ring (Figure S7, Supplementary Material). The superimposition (Figure 2, panel B) of the minimised conformers of **8d** extracted from MD simulations (*T* = 600 K, *ε* = 4*r*) revealed two possible orientations for the carbonyl group of the ester function at position 5, which is supposed to be involved in the catalytic process (energies are comprised within about 5 kcal mol^−1^).

**Figure 3.**
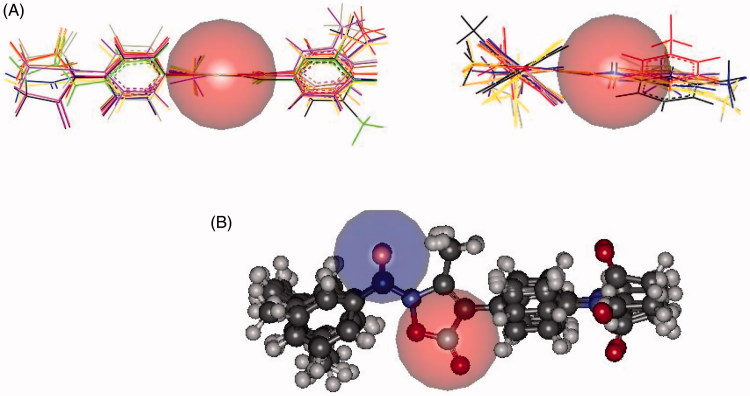
Panel (A): Accessibility of the carbonyl grouping involved in the catalytic process estimated by the dimension of a sphere (in red, *r* = 2.5 Å) centred on the carbon atom of **7d** (left) and **8d** (right). Panel (B): Accessibilities of the carbonyl groups in **7d** involved in the catalytic process and in H-bond interactions with the receptor points estimated by the dimension of a sphere centred on the carbon and oxygen atom (*r* = 2.5 Å, red and blue, respectively).

In both inhibitors, accessibility of the carbonyl group involved in the catalytic process was roughly estimated by the dimension of a sphere centred on the C = O carbon atom (*r* = 2.5 Å, in red in Figure 3, panel A). Similarly a sphere (*r* = 2.5 Å, in blue in Figure 3, panel B) centred on the oxygen atom, which could be involved in H-bond interactions with the receptor points, was used to assess the exposure to the environment of C = O in **7d**. Panel A of Figure 3 suggests a more crowded region about the site of the nucleophilic attack of **8d**, which could account for its lower activity compared to **7d** (in **8d**, the carbonyl group is directly bound to the phenyl ring and faces the pending arm at position 4). In addition, the almost cylindrical shape of **7d** caused, at least in principle, the carbonyl carbon atom to be attacked from both the side of the plane defined by the 5-membered ring (Figure S8, Supplementary Material). In contrast, only one side of the ester group in **8d** appeared to be easily accessible to the hydroxyl group of Ser195 (Figure S9, Supplementary Material). Finally, in both cases the terminal amide group appeared to be well exposed and thus prone for anchoring to the sub-pocket of the binding site.

Two different approaches were used to gain an idea of the interaction of **7d** and **8d** with HNE. First, docking of the inhibitors (two different starting conformations were considered) into the active site of HNE using the CDocker protocol (in vacuum, target temperature 300 K, 20 poses retained) and secondly, MD simulations on each inhibitor-HNE complex (in vacuum, simulation time 5 ns, *T* = 300K, with the atoms outside the binding sphere constrained to fixed points in space in order to save computational time). The structure of HNE complexed with a peptide chloromethyl ketone inhibitor was used for the docking study and MD simulations (1HNE^70^ entry of the Protein Data Bank). The binding site of HNE was defined as a sphere with a 12 Å radius centred at the centroid of the five-membered ring of the peptide chloromethyl ketone inhibitor which covered all the active site amino acids of the HNE enzyme. All water molecules and bound inhibitor were removed from the macromolecule and hydrogen atoms were added.

**Figure 4.**
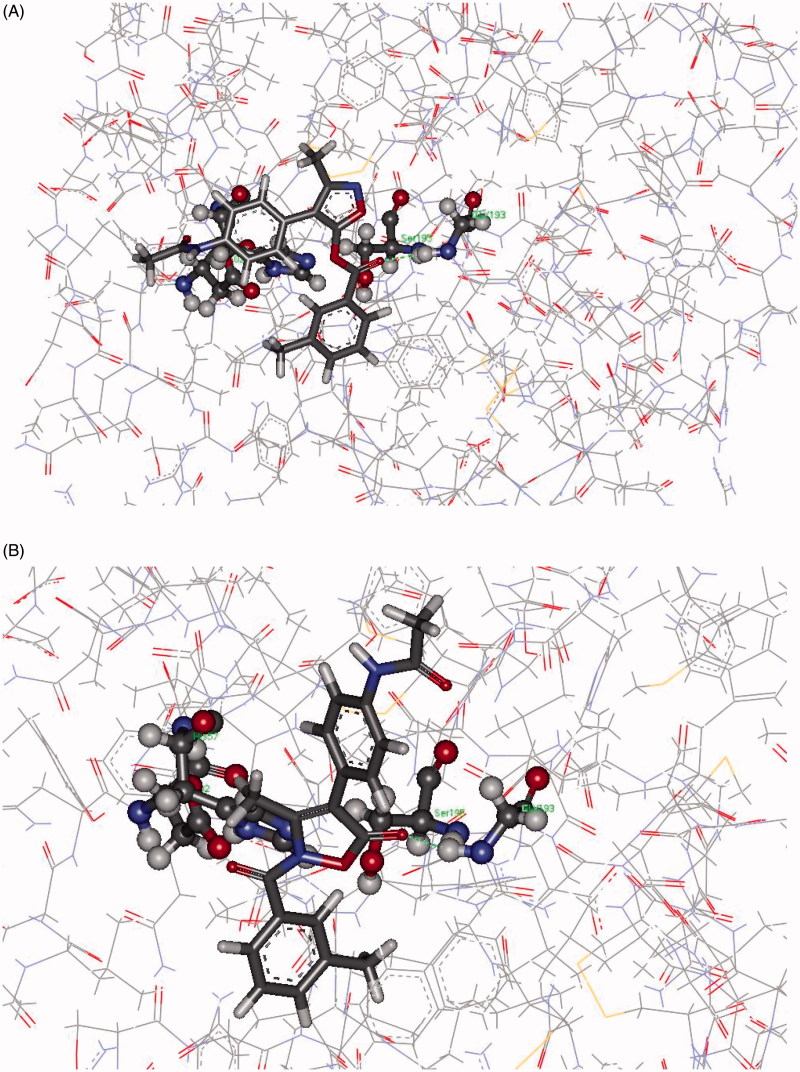
Panel (A): Pose from the CDocker protocol showing Gly193 involved as H-bond donor to the carbonyl function of **8d**. Panel (B): Pose from the CDocker protocol showing Gly193 involved as H-bond donor to 5-CO in **7d**.

Interaction energies from docking protocols on **7d** and **8d** with HNE did not significantly differ, and the two inhibitors showed comparable binding modes. In particular for **8d**, most of the saved poses featured Gly193 as the anchor group, and the H-bond interaction with this amino acid pushed the site of the nucleophilic attack quite distant from Ser195. However, a few poses show Gly193 involved as an H-bond donor to the ester function of **8d** and, in these cases, the distance HO(Ser195)…CO ranged from 3.3 to 4.8 Å (Figure 4, Panel A). The same amino acids are also involved in the interaction with **7d**. Anchoring of the terminal NHCO group to Ser195 *via* H-bonds pushed the CO at position 5 distant from the nucleophilic –OH of Ser195, while H-bonds between 5-CO and Gly193 brought the two partners of the nucleophilic attack a little bit closer (distances range from 4.3 to 5.1 Å, Figure 4, Panel B). However, the mean distance between the inhibitor-HNE reacting sites, as determined from the saved poses, was very long for both complexes (ca 7 Å).

**Figure 5.**
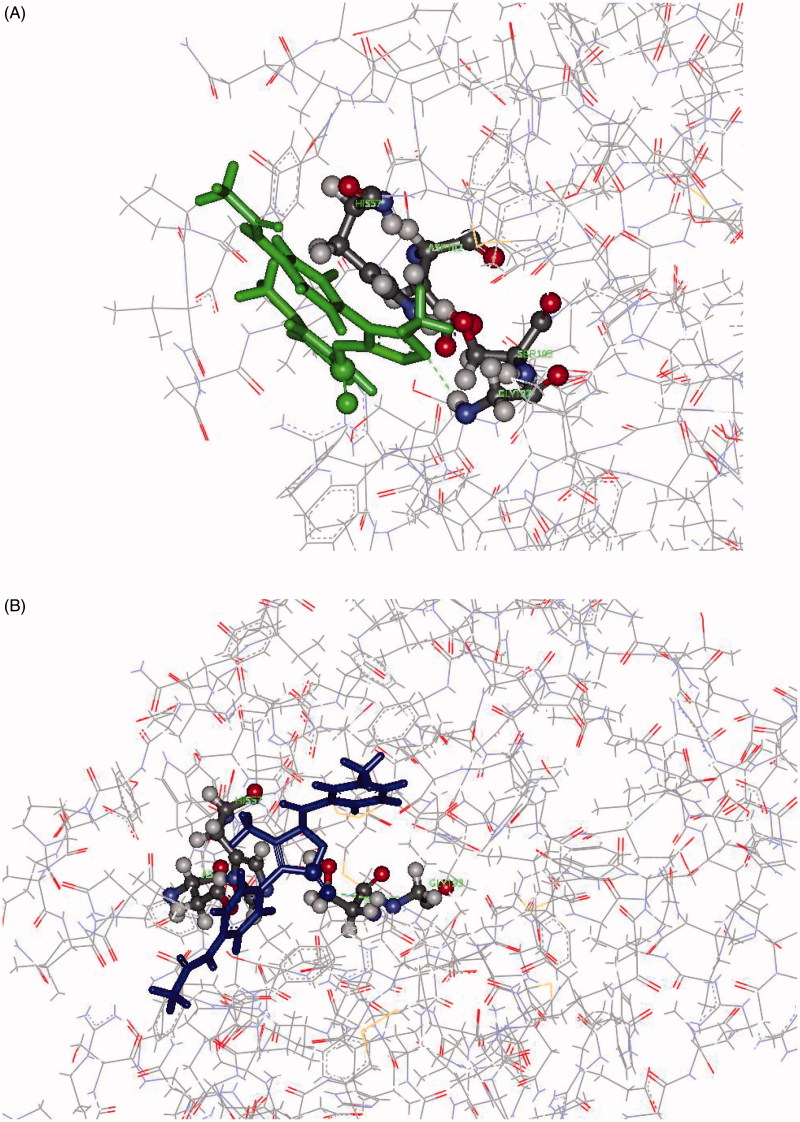
Panel (A): View of the **8d**-HNE adduct from MD showing the H-bond interaction between the nitrogen atom of the 5-membered ring acting as H-bond acceptor towards Gly193. Panel (B): View of the **7d**-HNE adduct from MD showing the H-bond interaction between the oxygen of 5-CO acting as H-bond acceptor towards Gly193.

In summary, docking results do not help to explain the different activities of the two inhibitors towards HNE. The fact that the CDocker protocol keeps the receptor rigid, while the inhibitor is allowed to flex during the refinement, could however significantly affect the results, and this is the reason why the ligand–receptor binding affinity was also assessed by MD. On the other hand, MD simulations confirmed the leading role of Gly193 as an anchoring group for both inhibitors. However, in contrast with the docking results, MD shows the 5-CO grouping of **7d** was significantly closer to the HNE reactive site (distance less than 4.5 Å in a large fraction of the snapshot conformations) with respect to the corresponding active site of **8d** (*d* < 5 Å in few of the sampled conformations). In contrast, a comparison of the total number of the intermolecular DH…A contacts (distances less than 2.5 Å) shows a greater propensity for **8d** to form H-bond interactions compared to **7d**. In addition, while multiple intermolecular H-bonds were present for **8d**, most of the snapshot conformations of **7d** exhibited only one H-bond interaction. In other words, the net of intermolecular H-bonds that involves **8d** keeps the molecule quite distant from Ser195. Finally, it is noteworthy that in the HNE complexes with **8d** featuring closer distances between the active sites, the heteroatoms of the 5-membered ring acted as H-bond acceptors both towards the Ser195 –OH group and Gly193. As a result of these interactions, the relative 3 D arrangement of **8d** in the HNE active site does not appear to be propitious for approach of the nucleophile of Ser195 (see for example Figure 5, Panel A). In contrast, the H-bond between 5-CO and Gly193, which in most cases held together the **7d**/HNE adduct, results in a favourable 3 D arrangement of the reacting partners (see for example Figure 5, panel B) for nucleophilic attack.

### Stability and kinetic features

The most potent isoxazolones with IC_50_ < 100 nM and their ester analogs were further evaluated for chemical stability in aqueous buffer using spectrophotometry to detect compound hydrolysis. The compounds had *t*_1/2_ values from 2.9 to 9.6 h for spontaneous hydrolysis, indicating that the amides were more stable than the esters in the corresponding pairs of **7a/8a**, **7d/8d**, and **7e/8e** (Table 4). In general, the isoxazolones were more stable than our previously described HNE inhibitors with cinnolinone^39^, N-benzoylindazole^36^^,^^37^, and N-benzoylpyrazole scaffolds^71^.

**Table 4.** Half-life (*t*_1/2_) for the spontaneous hydrolysis of selected derivatives.

Comp.	*t*_1/2_ (h)	Absorbance (nm)^a^
**4a**	4.1	330
**4b**	8.3	290
**7a**	7.7	340
**8a**	5.3	320
**7d**	3.9	340
**8d**	2.9	330
**7e**	9.6	320
**8e**	5.6	280

a Absorption used for monitoring spontaneous hydrolysis.

**Figure 6.**
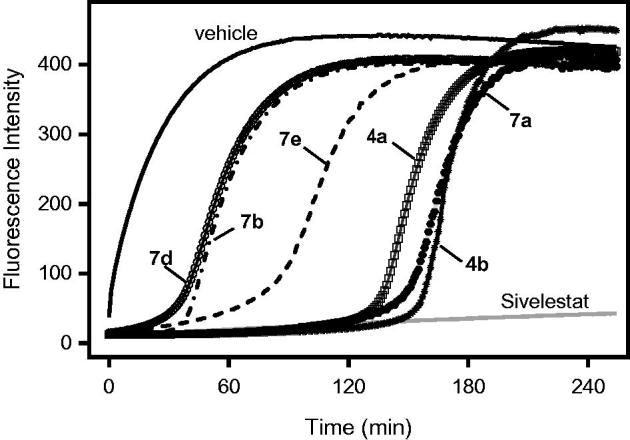
Evaluation of HNE inhibition by representative isoxazolones and Sivelestat over extended periods of time. HNE was incubated with the indicated compounds (8 µM), and kinetic curves monitoring substrate cleavage catalysed by HNE over time are shown. Representative curves are from two independent experiments.

The most potent isoxazolones were also selected for evaluation of the reversibility of HNE inhibition over time. As shown in Figure 6, HNE inhibition was rapidly (∼30 min) reversed for compounds **7d** and **7b**. The inhibition was maximal for up to 60 min with compound **7e** and >120 min for the other tested compounds (**4a**, **4b**, and **7a**). However, inhibition by the compounds was eventually reversed, and full recovery of HNE activity was observed by 4 h after treatment with 8 µM of the compound (e.g. see Figure 6).

**Figure 7.**
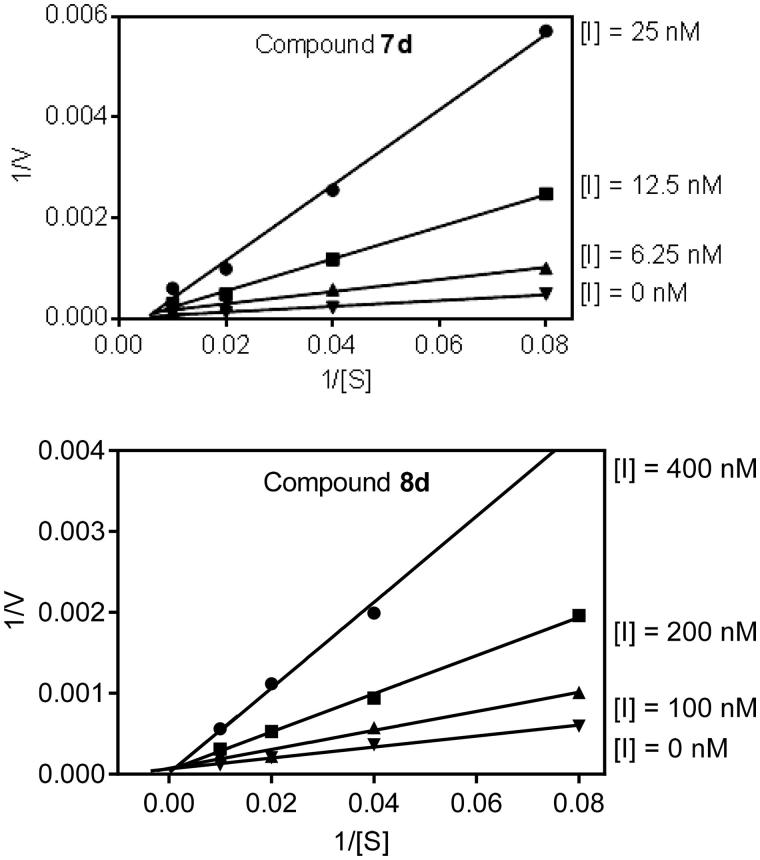
Kinetics of HNE inhibition by compounds **7d** and **8d**. Representative double reciprocal Lineweaver–Burk plots are shown from three independent experiments.

To better understand the mechanism of action of these isoxazolone HNE inhibitors, we performed kinetic experiments. As shown in Figure 7, the representative double-reciprocal Lineweaver–Burk plot of fluorogenic substrate hydrolysis by HNE in the absence and presence of compounds **7d** and **8d** indicates that these compounds are competitive HNE inhibitors.

## Conclusions

In the present study, we report a new series of isoxazolones as potent HNE inhibitors, confirming our previous results^40^, which indicated this nucleus as an appropriate scaffold for this target. The most potent compounds had a methyl group at position 3 and a (substituted)phenyl ring at position 4, and the higher HNE inhibitory activity was found for compound **7a** (IC_50_* =* 20 nM). In addition to the 2-NCO derivatives, a number of 5-NCO compounds were obtained, although with lower activity than the corresponding amide. Studies of chemical stability in aqueous buffer indicated that amides were generally more stable than esters, while kinetic experiments confirmed that both amides and esters were competitive HNE inhibitors. Docking and molecular dynamics studies are the different approaches used to understand the interactions of the two isomers **7d** and **8d** with HNE. Both studies highlight the fundamental role of Gly193 as anchoring group for both the inhibitors, but the MD simulations help us to explain the difference in inhibitory activity between the inhibitors **7d** and **8d**. The ester isomer **8d** shows a greater propensity to form H-bond interactions with respect to **7d** and as a result the molecule is quite distant from the Ser195, the amino acid responsible for the nucleophilic attack. While the amide isomer **7b** appears more mobile within the active site of HNE, being held in place by single H-bond interactions (vs. multiple in **8d**), which leads the C = O at position 5 to obtain a favourable orientation for the nucleophilic attack by Ser195.

## Supplementary Material

Supplemental Material
