# Supplementary material for: Synthesis, biological evaluation, and molecular modelling studies of potent human neutrophil elastase (HNE) inhibitors
Source: J Enzyme Inhib Med Chem. 2018 Jul 4;33(1):1108–24. doi: 10.1080/14756366.2018.1480615 (PMC6032016; doi:10.1080/14756366.2018.1480615)
Supplement: Supplemental Material [file IENZ_A_1480615_SM6644.pdf]

SUPPLEMENTAL MATERIAL FOR

Synthesis, biological evaluation, and molecular modeling studies of potent human neutrophil elastase (HNE) inhibitors

Maria Paola Giovannoni[a], *Igor A. Schepetkin*[b], *Mark T. Quinn*[b], *Niccolò Cantini*[a], *Letizia Crocetti*[a*], *Gabriella Guerrini*[a], *Antonella Iacovone*[a], *Paola Paoli*[c], *Patrizia Rossi*[c], *Gianluca Bartolucci*[a], *Marta Menicatti*[a], *Claudia Vergelli*[a]

[a]NEUROFARBA, Pharmaceutical and Nutraceutical Section, University of Florence, Via Ugo Schiff 6, 50019 Sesto Fiorentino, Italy.

[b]Department of Microbiology and Immunology, Montana State University, Bozeman, MT 59717, USA.

[c]Department of Industrial Engineering, University of Florence, Via Santa Marta 3, 50139 Florence, Italy.

[*]Corresponding Author

Letizia Crocetti

Dipartimento di NEUROFARBA

Via Ugo Schiff 6

Sesto Fiorentino 50019 Firenze

Tel +39-055-4573683

E-mail letizia.crocetti@unifi.it

Table of contents

1. ^{1}H-NMR, ^{13}C-NMR, HSQC, HMBC and NOESY spectra of isomers **7a/8a, 7b/8b** and **7d/8d**.
2. **Figure S1**. Schematic drawing of **7d** and **8d** showing the torsion angles defining the molecular conformation monitored during MD simulations (numbers identify the set of atoms which define a given torsion).
3. **Figure S2**. Dihedral angle τ_1-τ_2 distribution for **7d** during MD simulation (T=600K, ε=4r).
4. **Figure S3**. Dihedral angle τ_3- τ_5 distribution for **7d** during MD simulation (T=600K, ε=4r).
5. **Figure S4**. View of the lowest energy conformer of **7d** as found from MD simulations (T=600K, ε=4r).
6. **Figure S5**. Dihedral angle τ_1- τ_3 distribution for **8d** during MD simulation (T=600K, ε=4r).
7. **Figure S6**. Dihedral angle τ_4- τ_6 distribution for **8d** during MD simulation (T=600K, ε=4r).
8. **Figure S7**. View of the lowest energy conformer of **8d** as found from MD simulations (T=600K, ε=4r).
9. **Figure S8**. View of the lowest energy conformer of **7d** as found from QC (B3LYP functional).
10. **Figure S9**. View of the lowest energy conformer of **8d** as found from QC (B3LYP functional).
11. Elemental analysis (**Table S1**)

1H NMR compound 7a

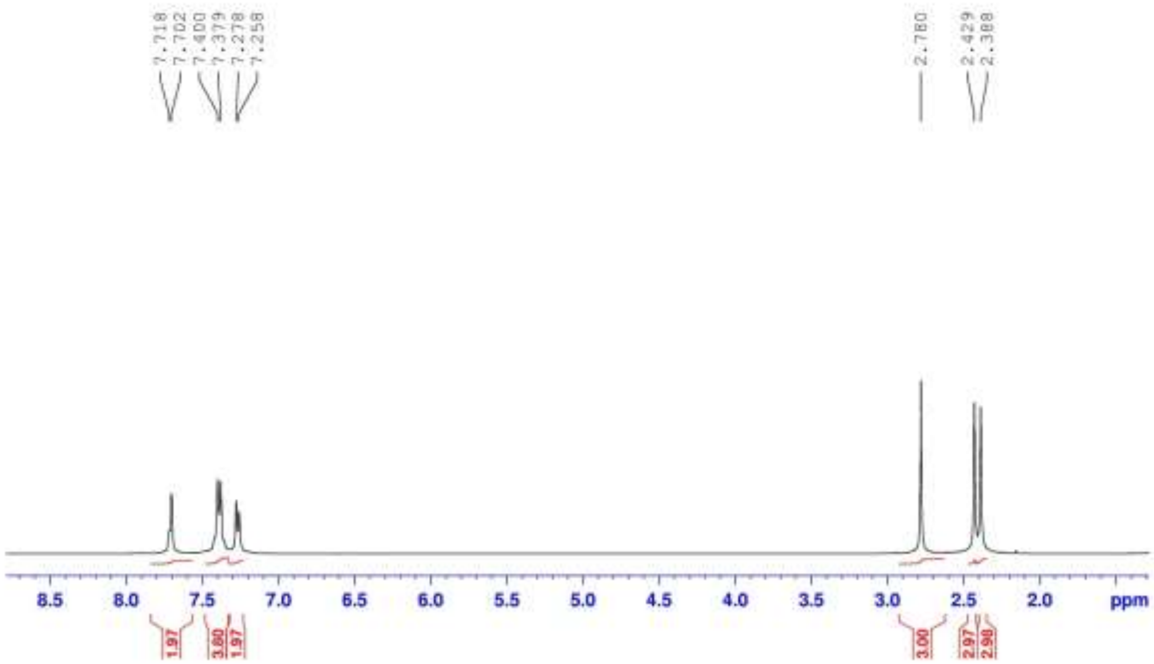

13C NMR compound 7a

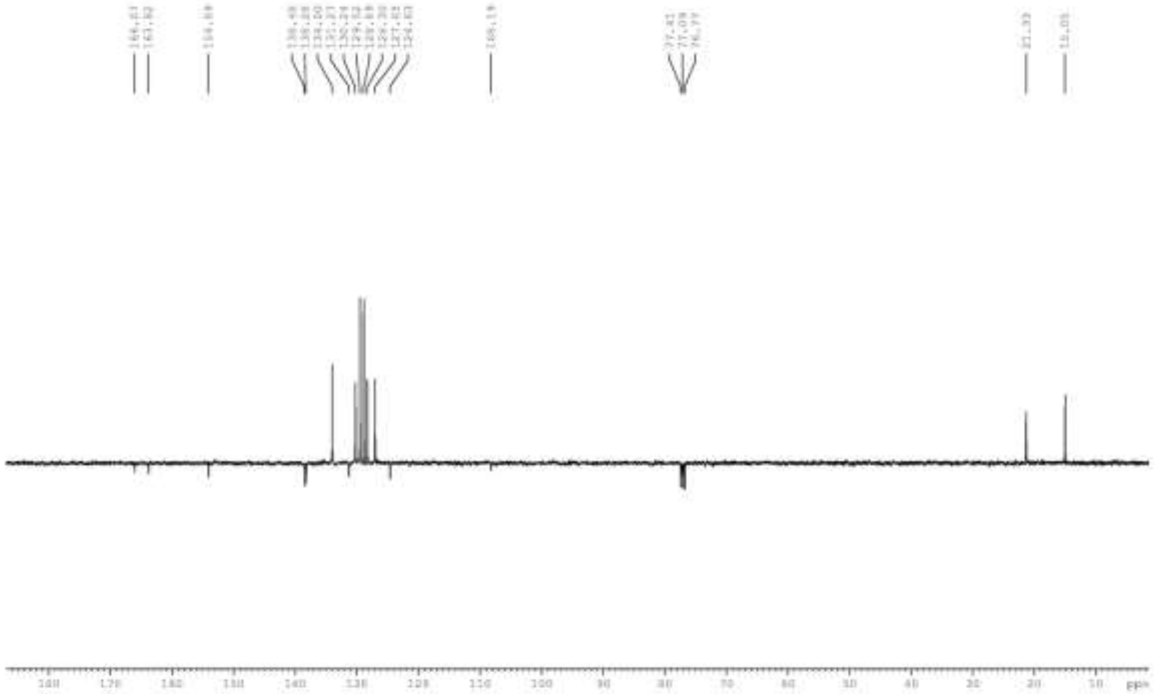

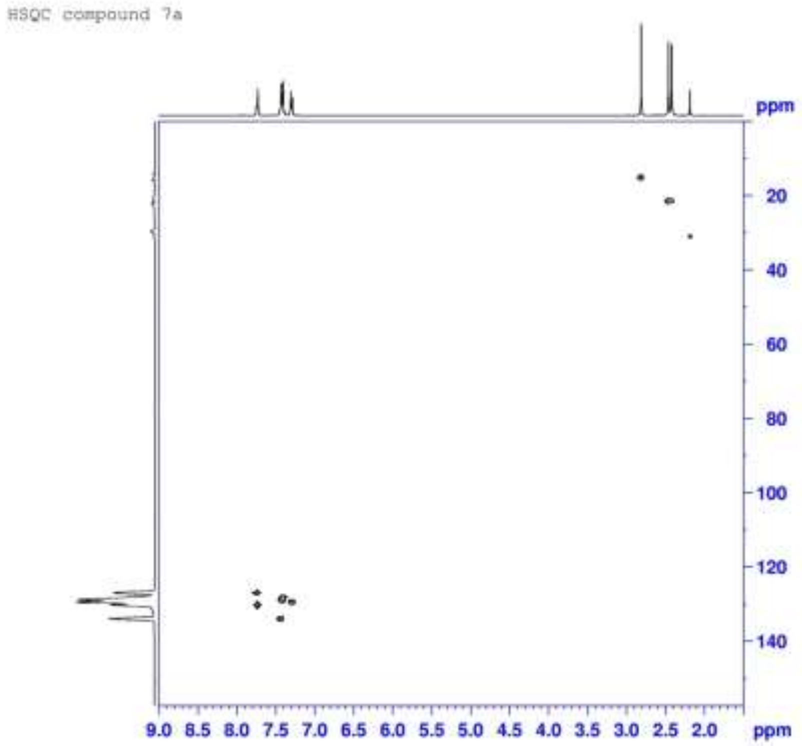
HSQC compound 7a
ppm
20
40
60
80
100
120
140
9.0 8.5 8.0 7.5 7.0 6.5 6.0 5.5 5.0 4.5 4.0 3.5 3.0 2.5 2.0
ppm

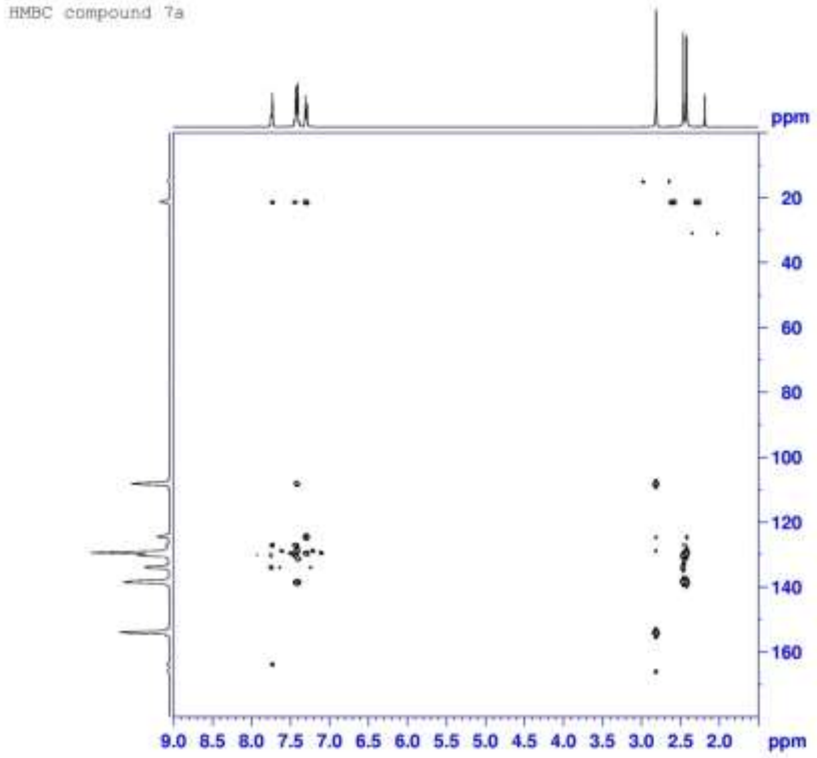
HMBC compound 7a
ppm
20
40
60
80
100
120
140
160
9.0 8.5 8.0 7.5 7.0 6.5 6.0 5.5 5.0 4.5 4.0 3.5 3.0 2.5 2.0
ppm

NOESY compound 7a

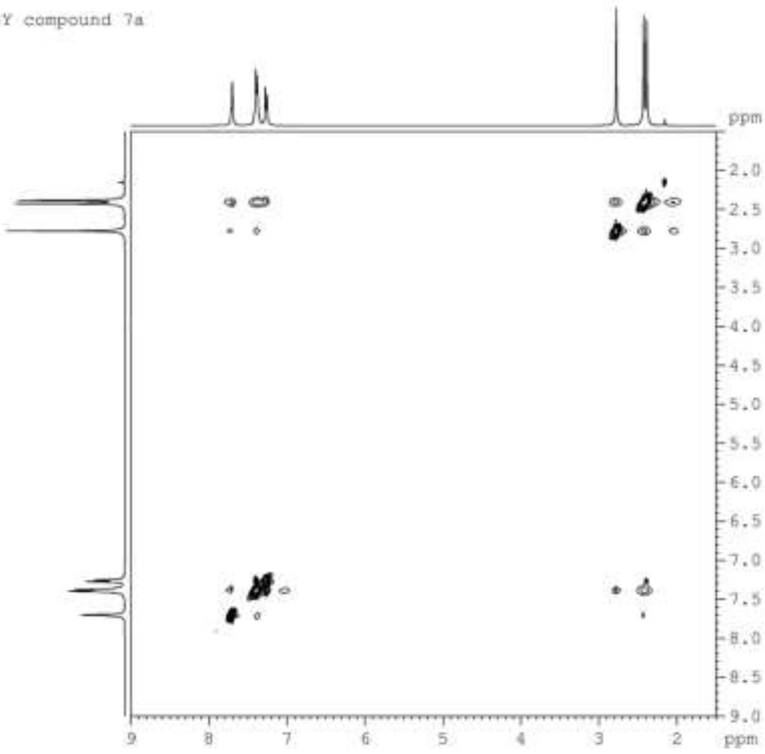

1H NMR compound 8a

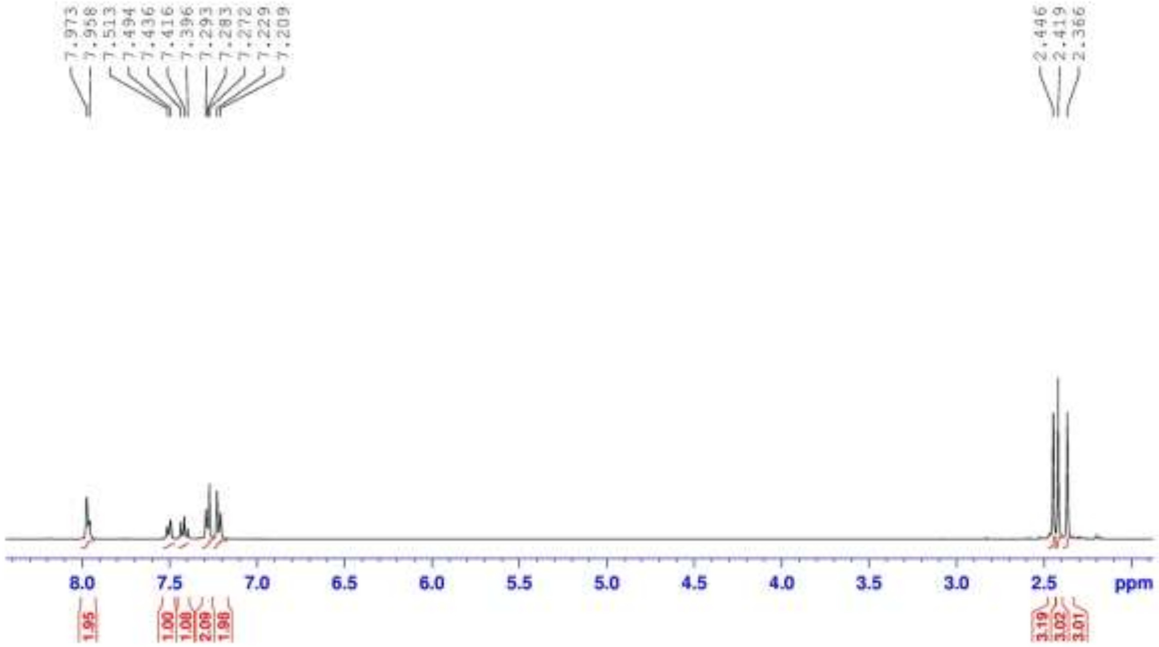

13C NMR compound 8a

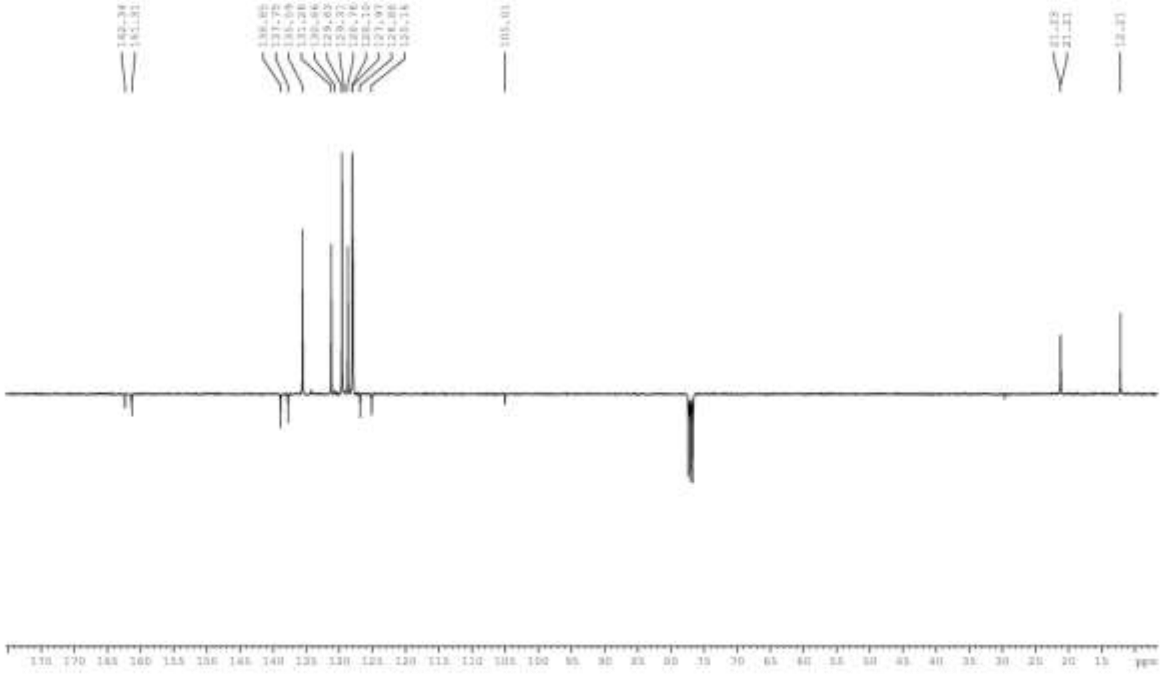

HSQC compound 8a

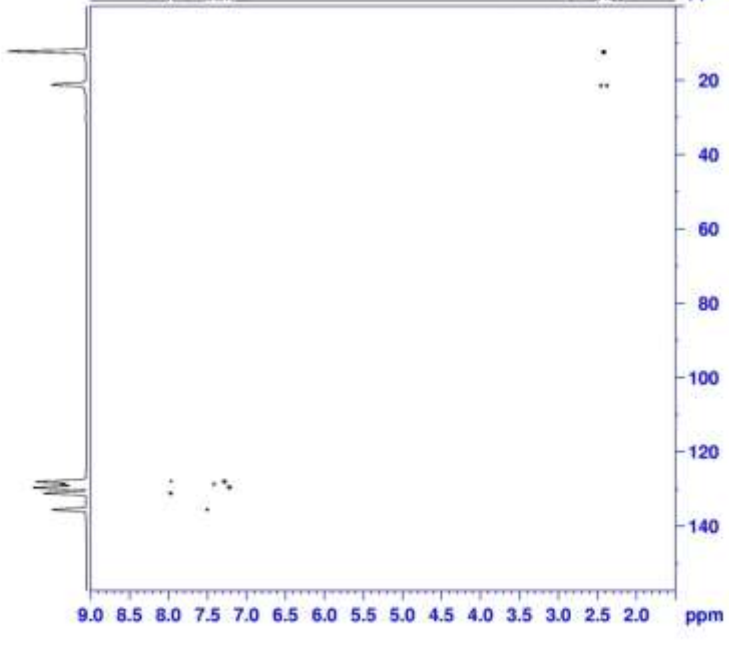

HMBC compound 8a

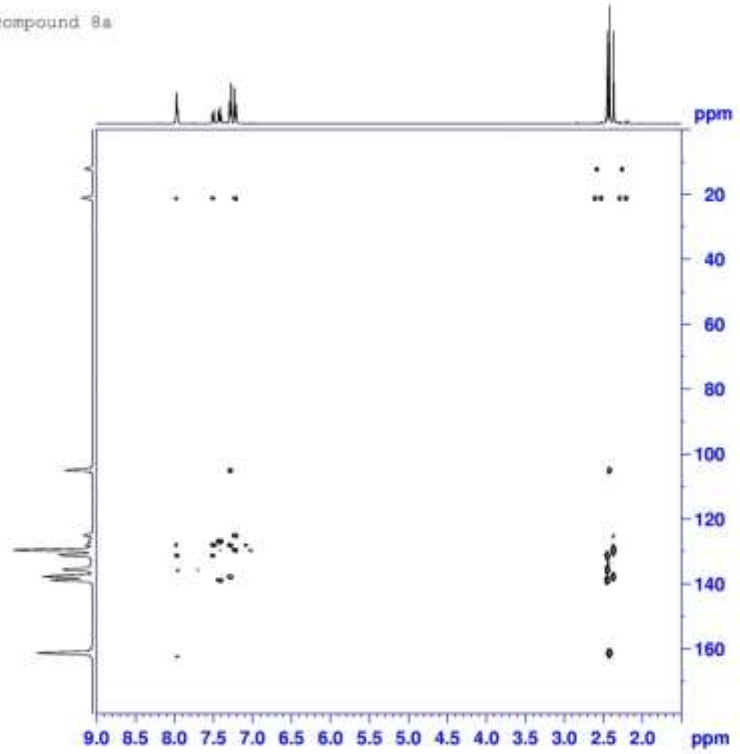

NOESY compound 8a

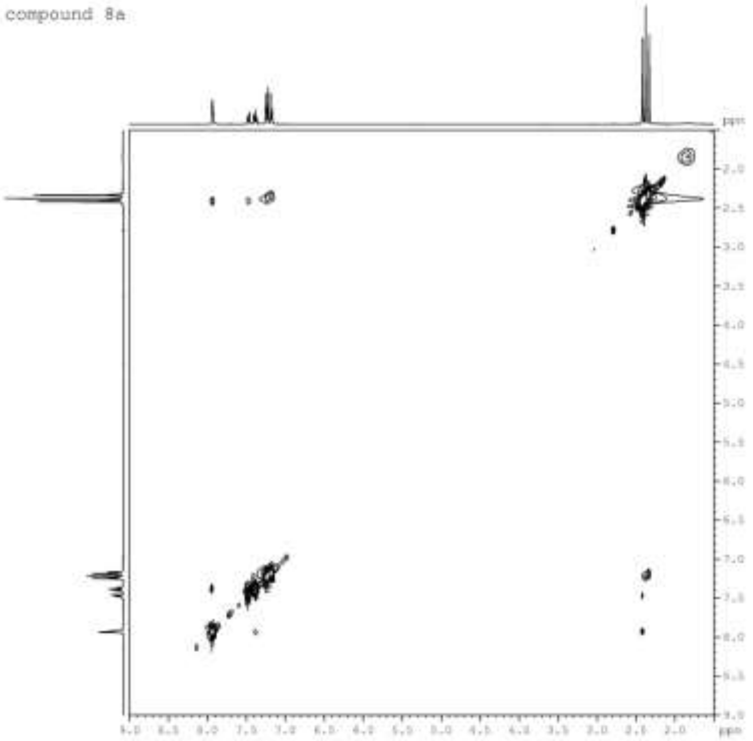

1H NMR compound 7b

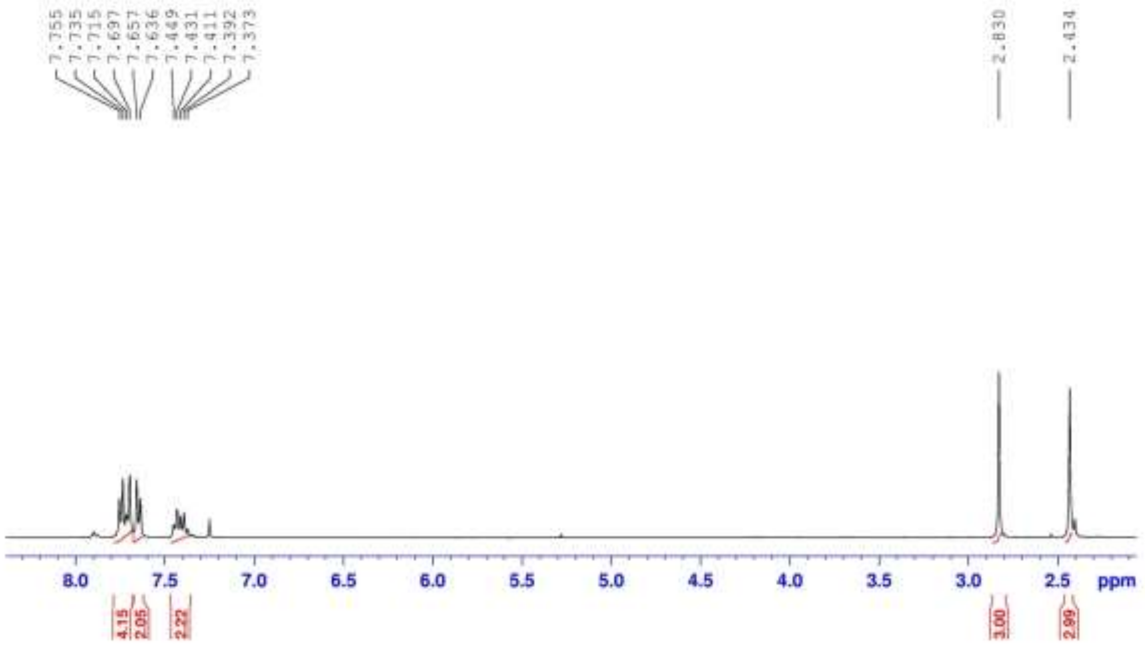

13C NMR compound 7b

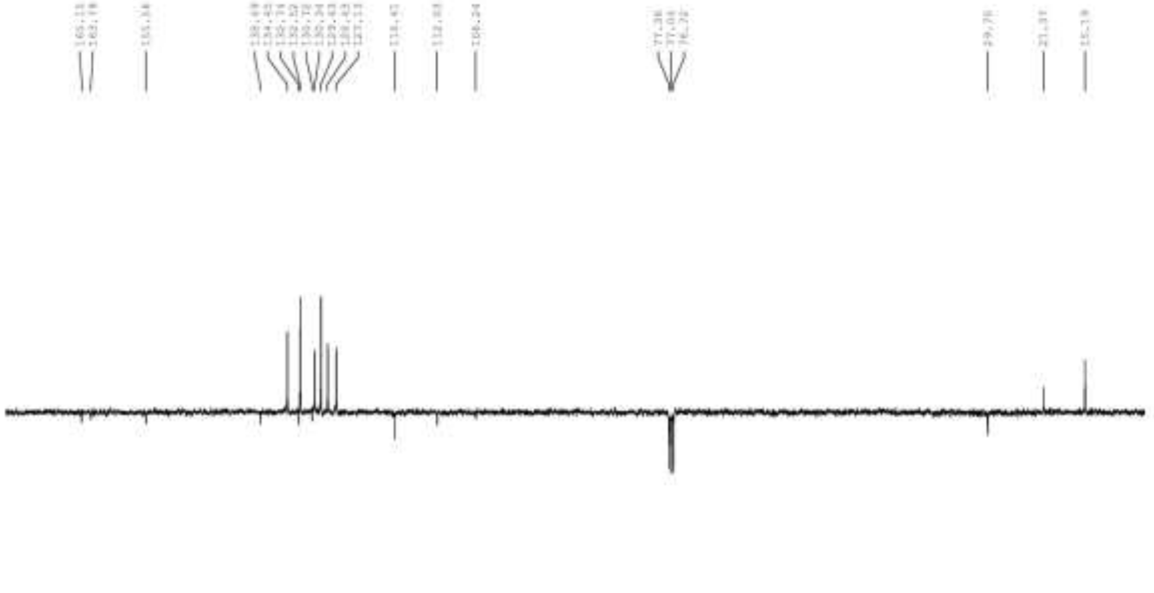

HSQC compound 7b

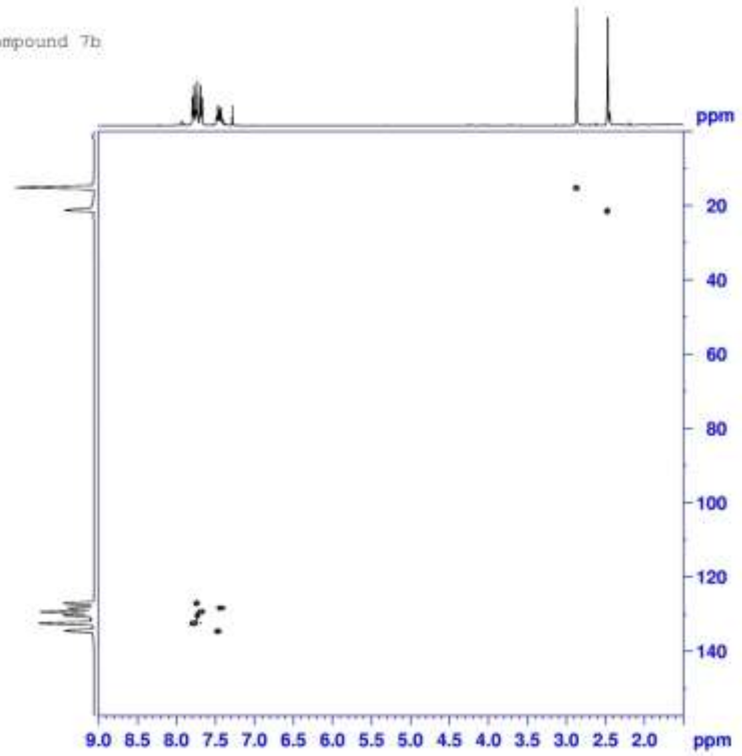

HMBC compound 7b

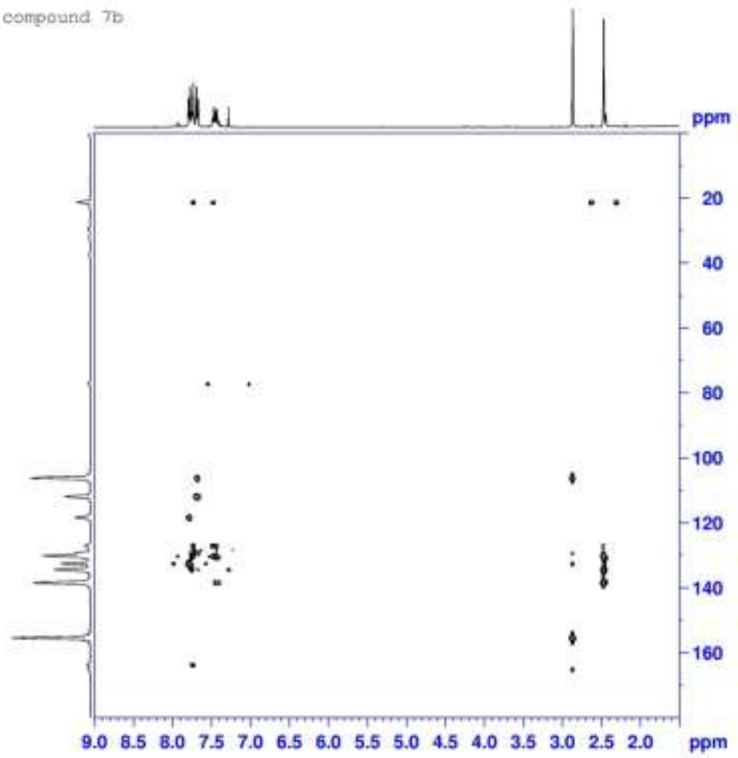

NOESY compound 7b

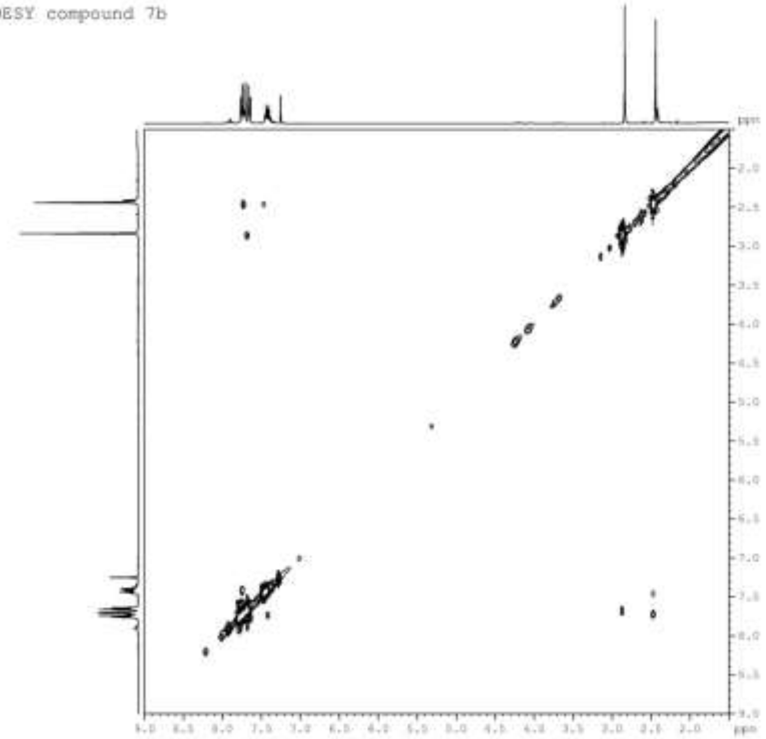

1H NMR compound 8b

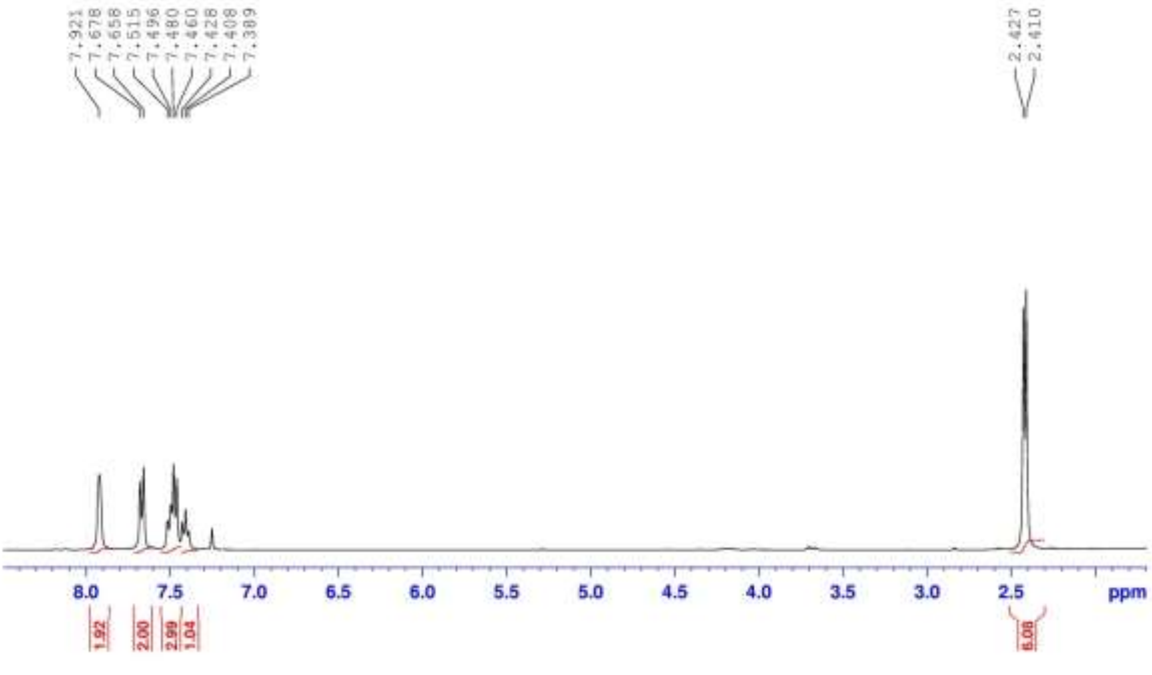

13C NMR compound 8b

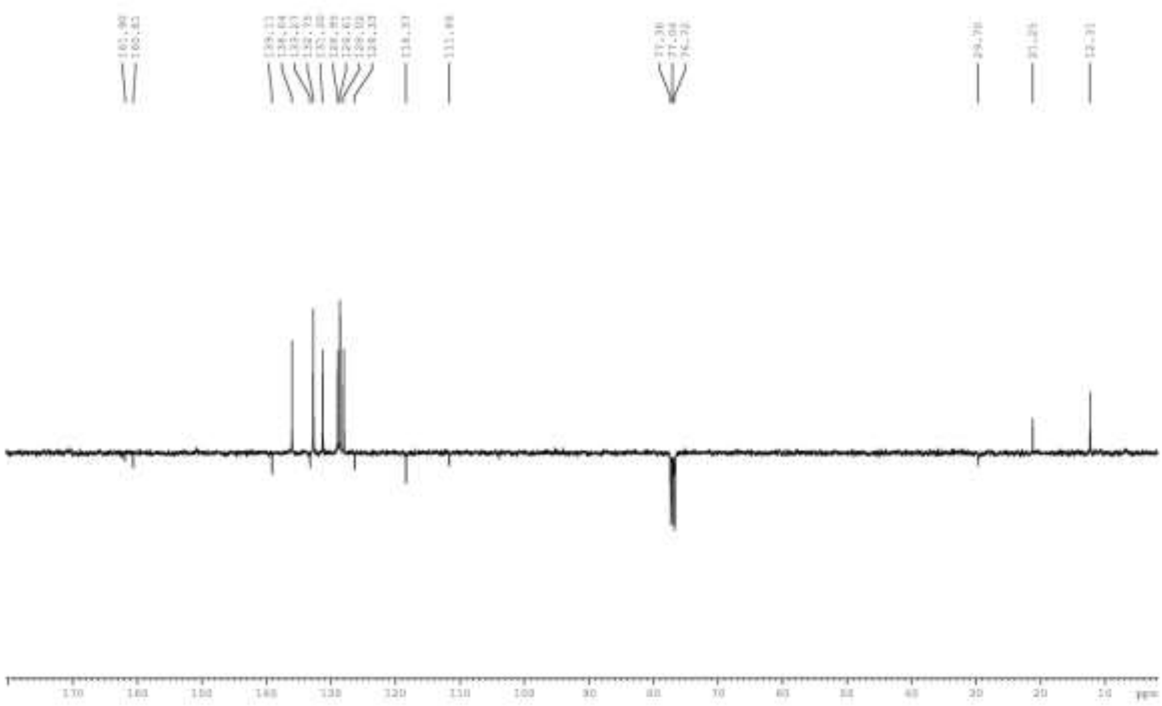

HSQC compound 8b

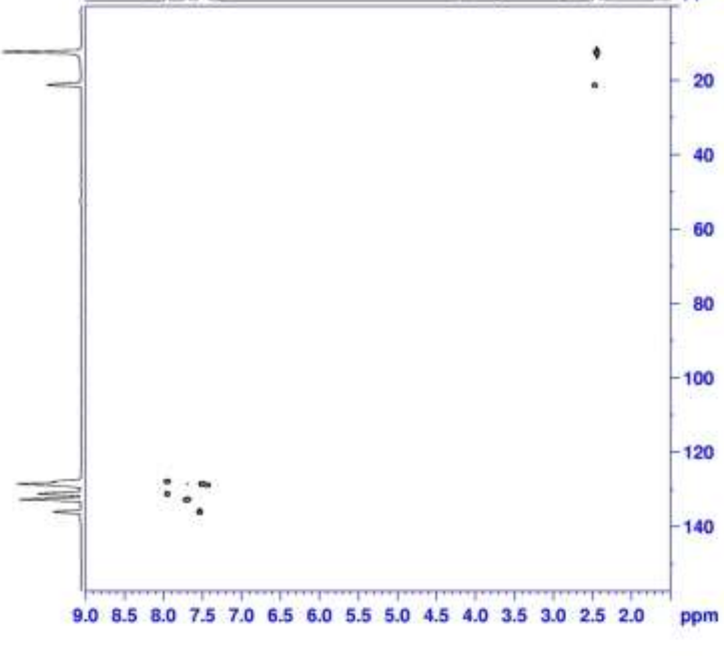

HMBC compound 8b

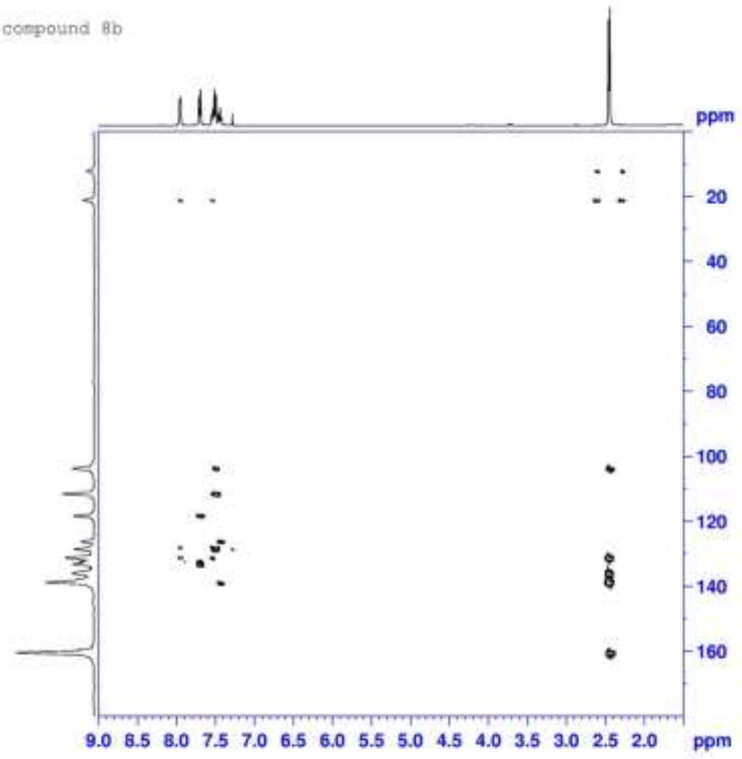

NOESY compound 8b

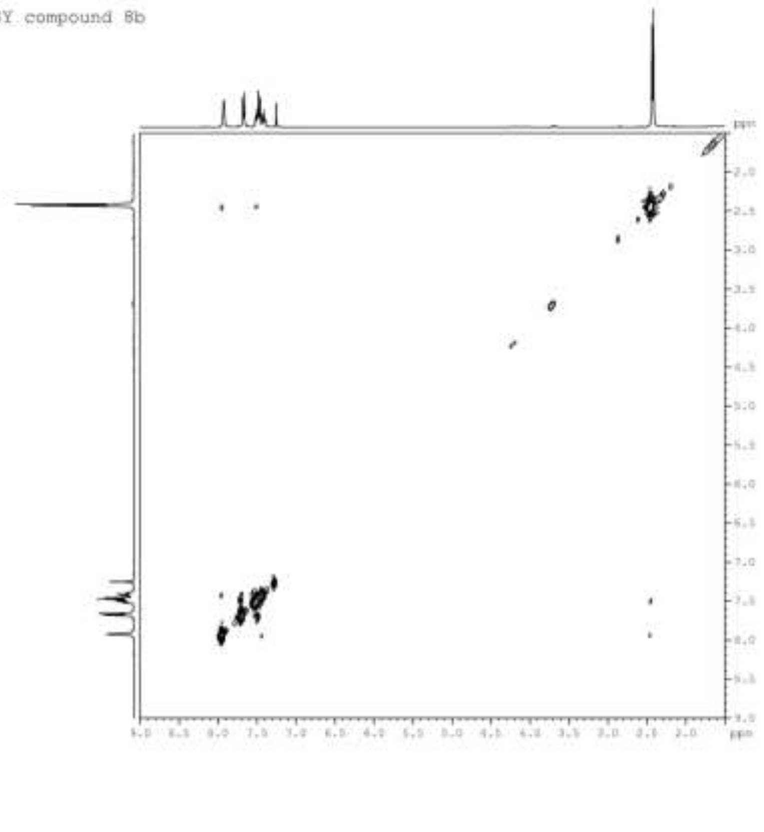

1H NMR compound 7d

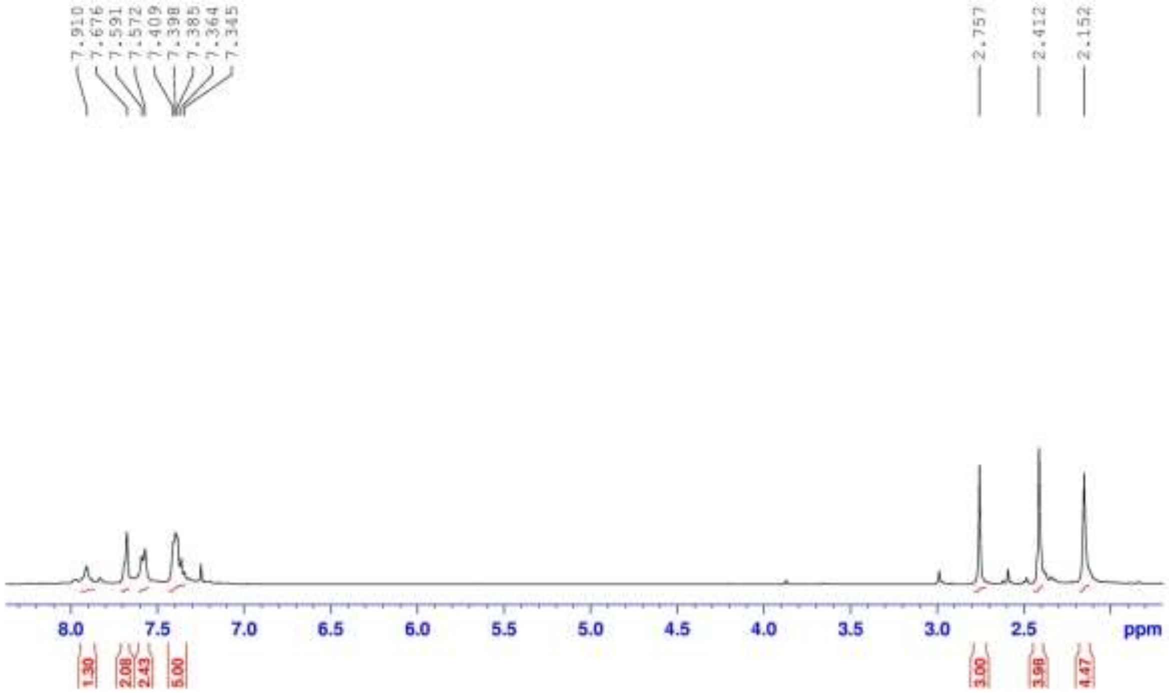

13C NMR compound 7d

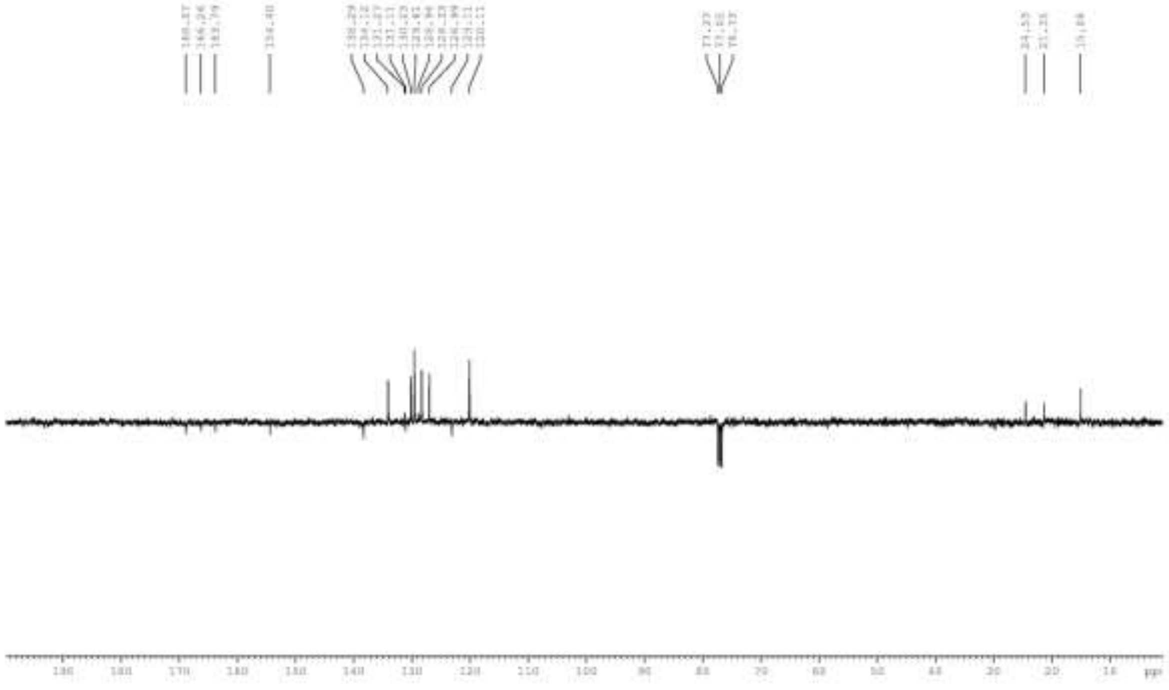

HSQC compound 7d

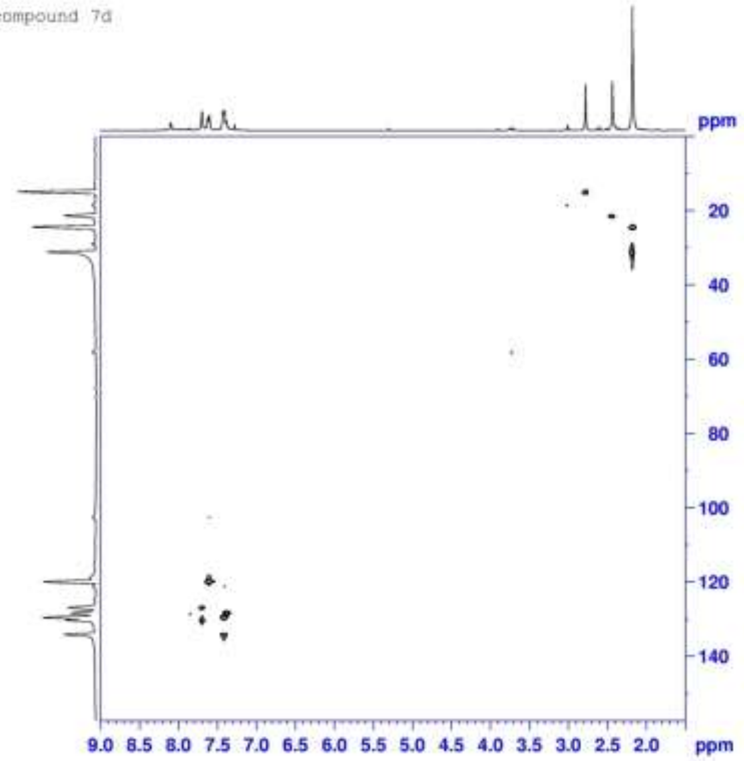

HMBC compound 7d

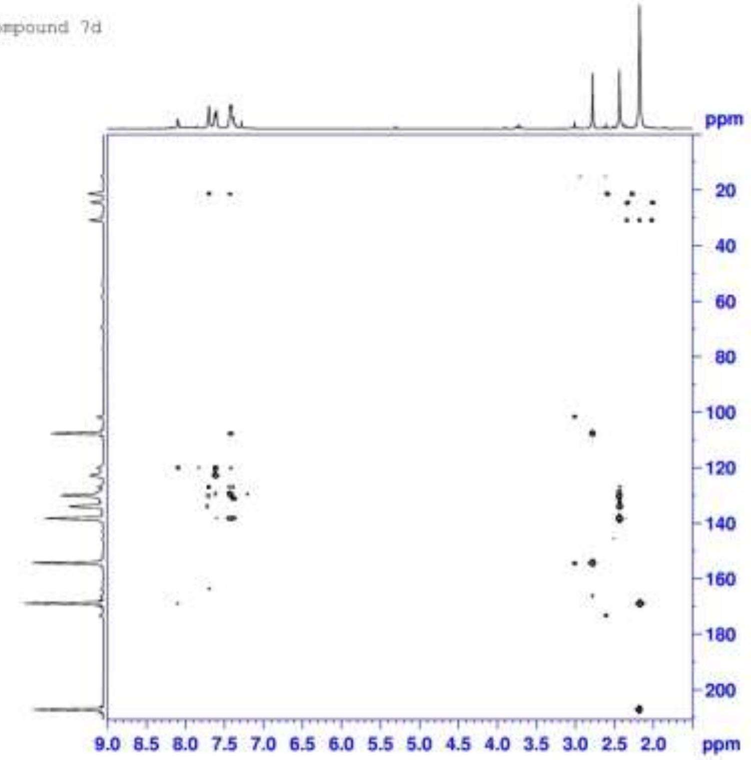

NOESY compound 7d

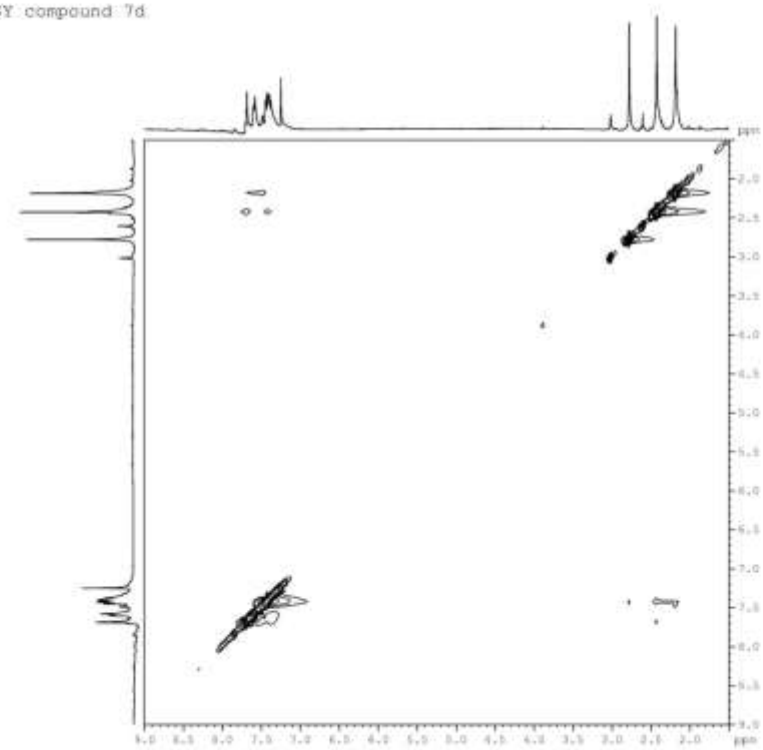

1H NMR compound 8d

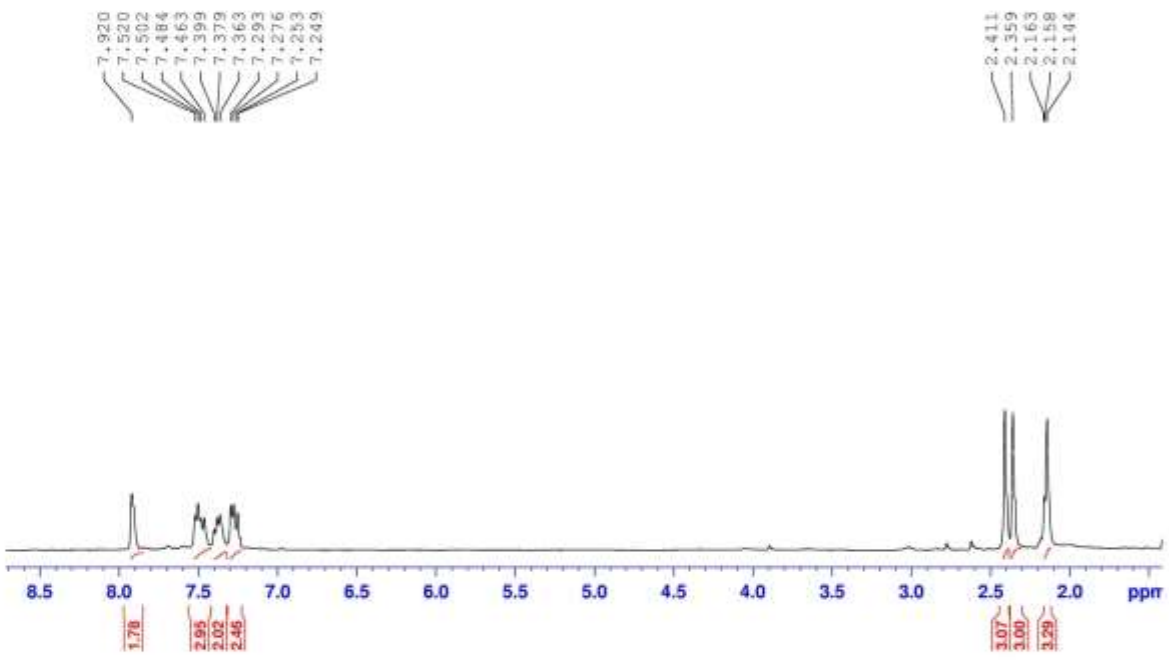

13C NMR compound 8d

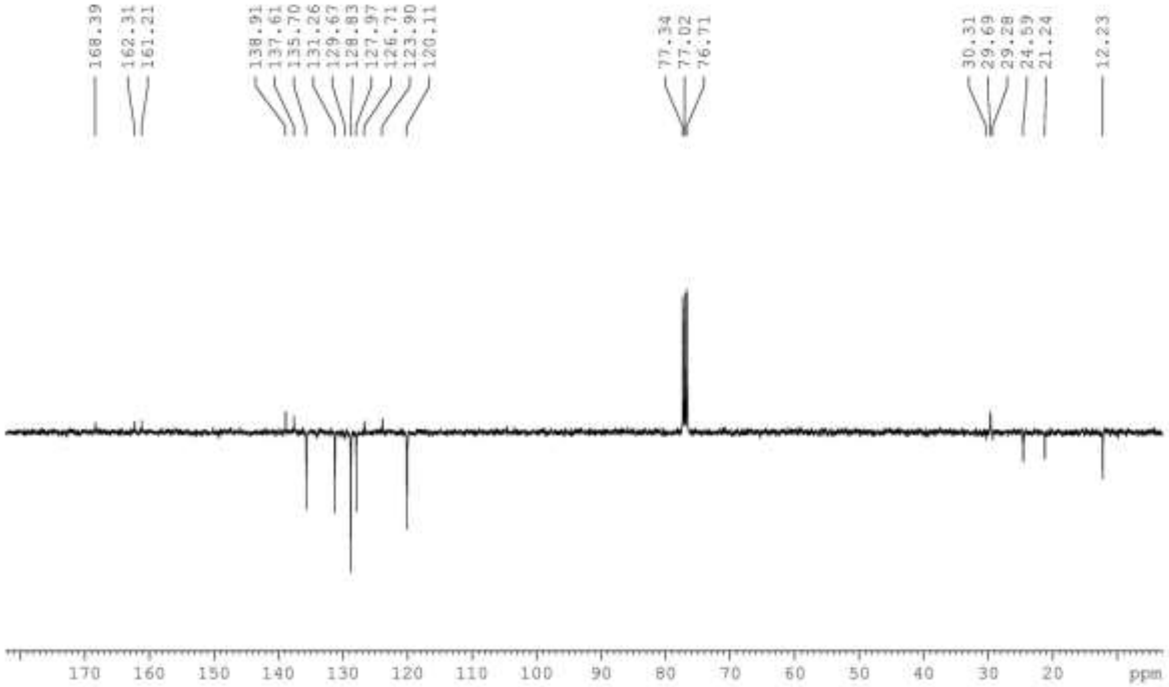

HSQC compound 8d

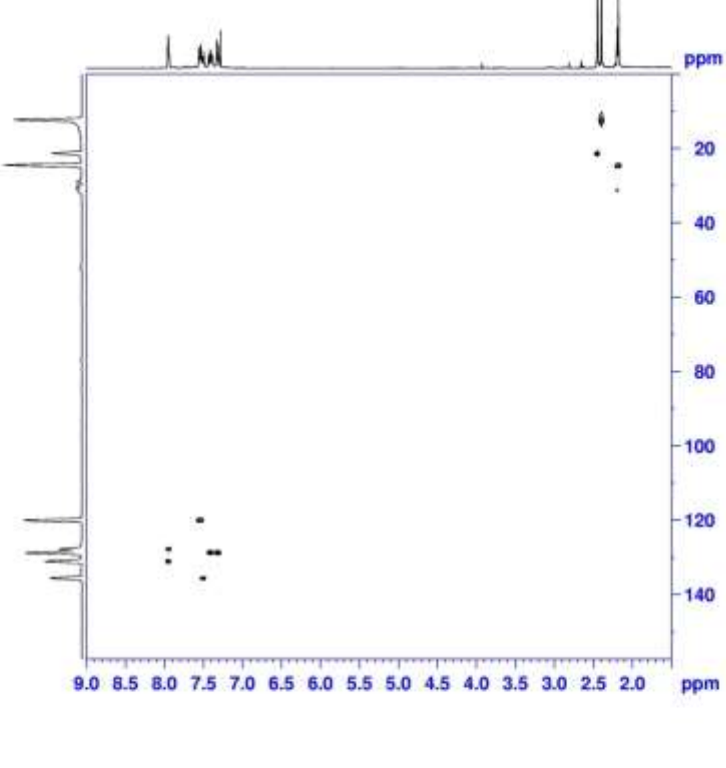

HMBC compound 8d

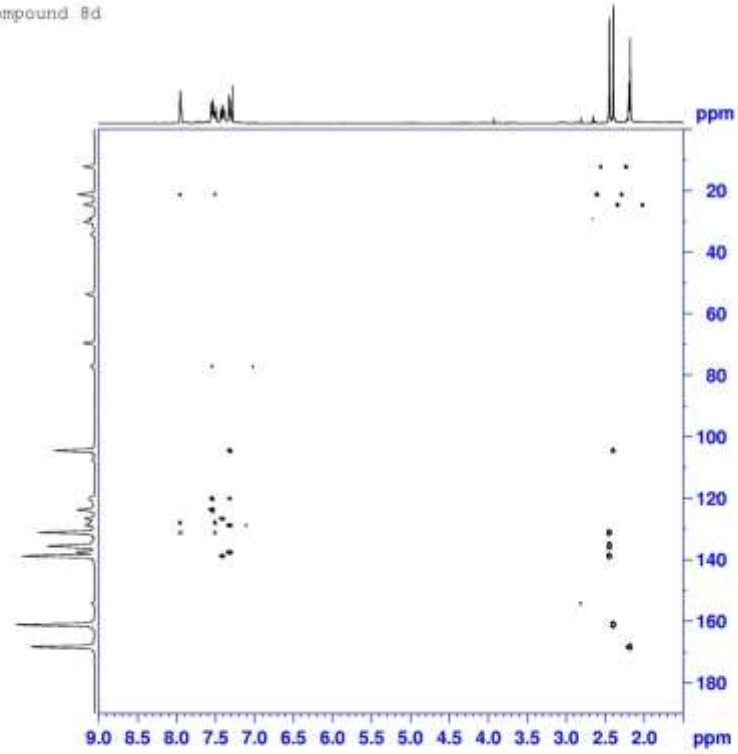

NOESY compound 8d

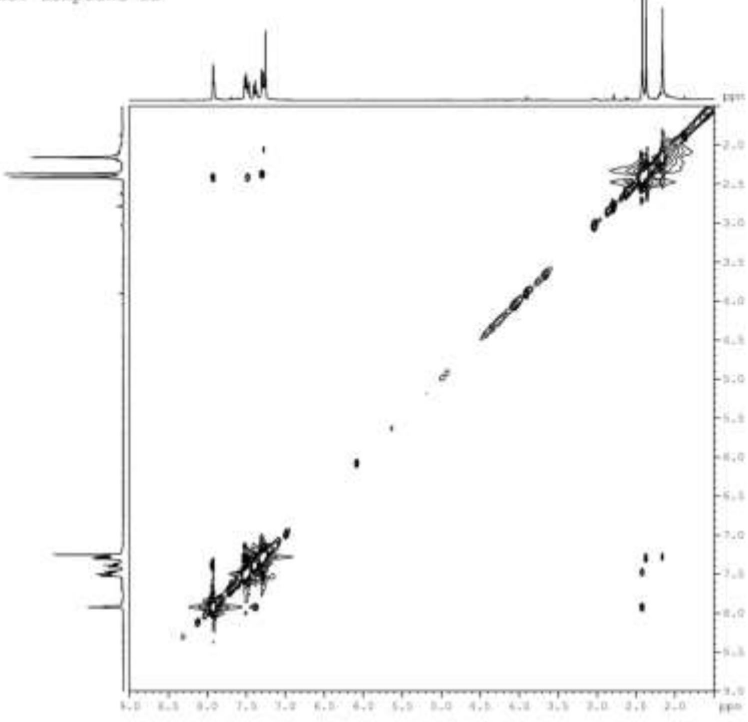

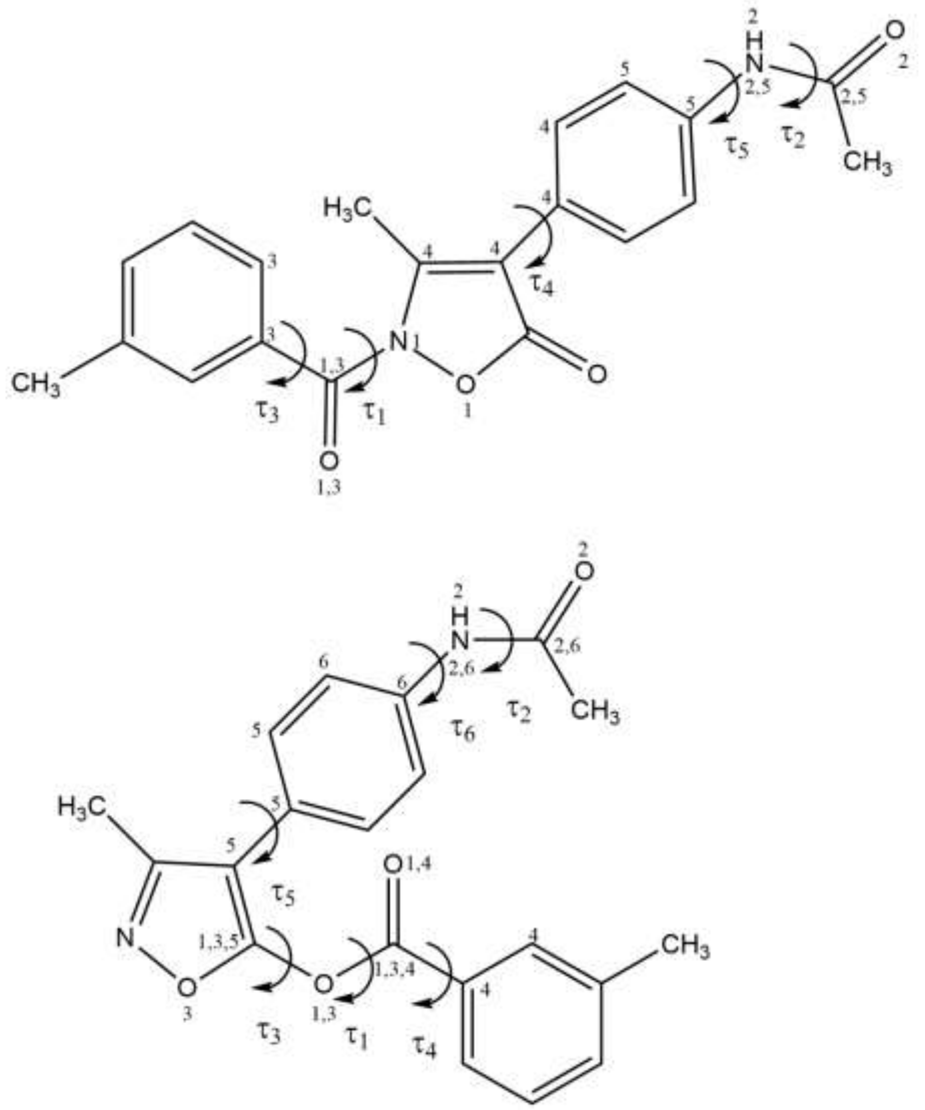

Fig. S1. Schematic drawing of **7d** and **8d** showing the torsion angles defining the molecular conformation monitored during MD simulations (numbers identify the set of atoms which define a given torsion).

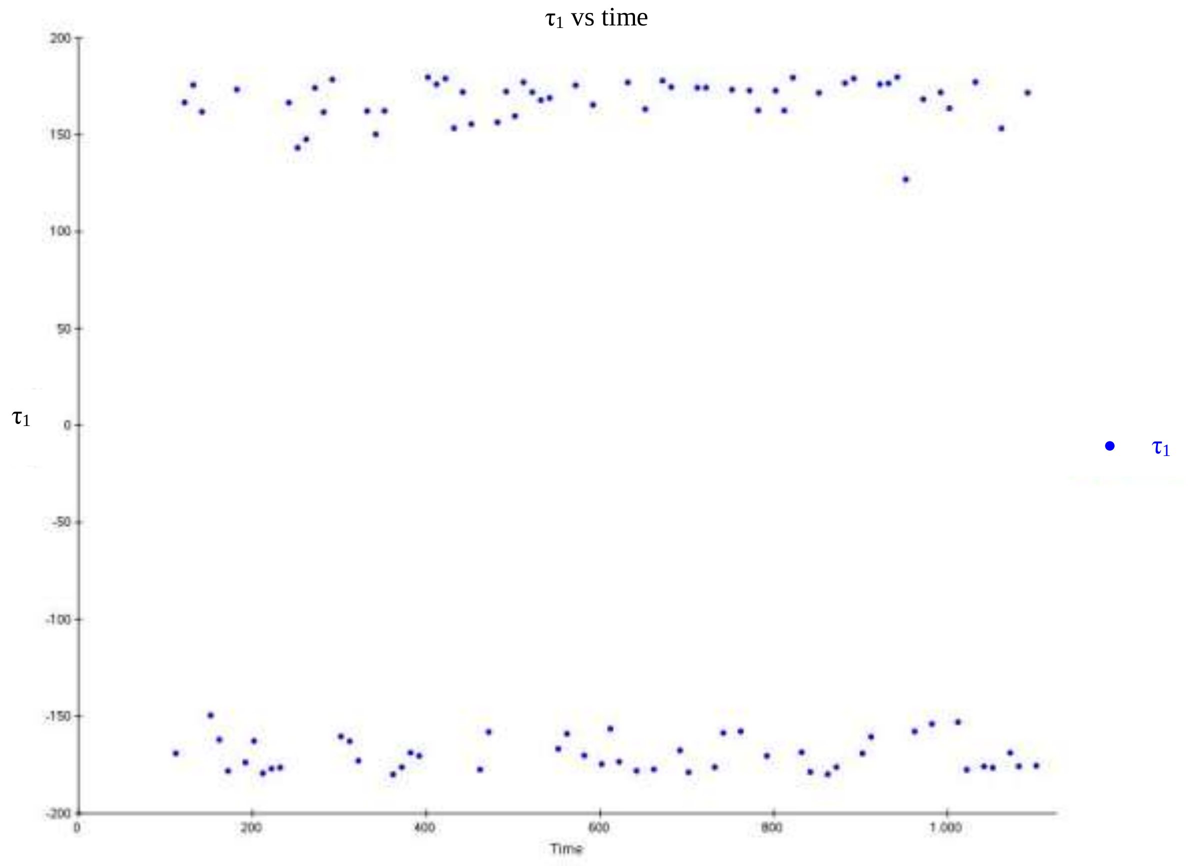

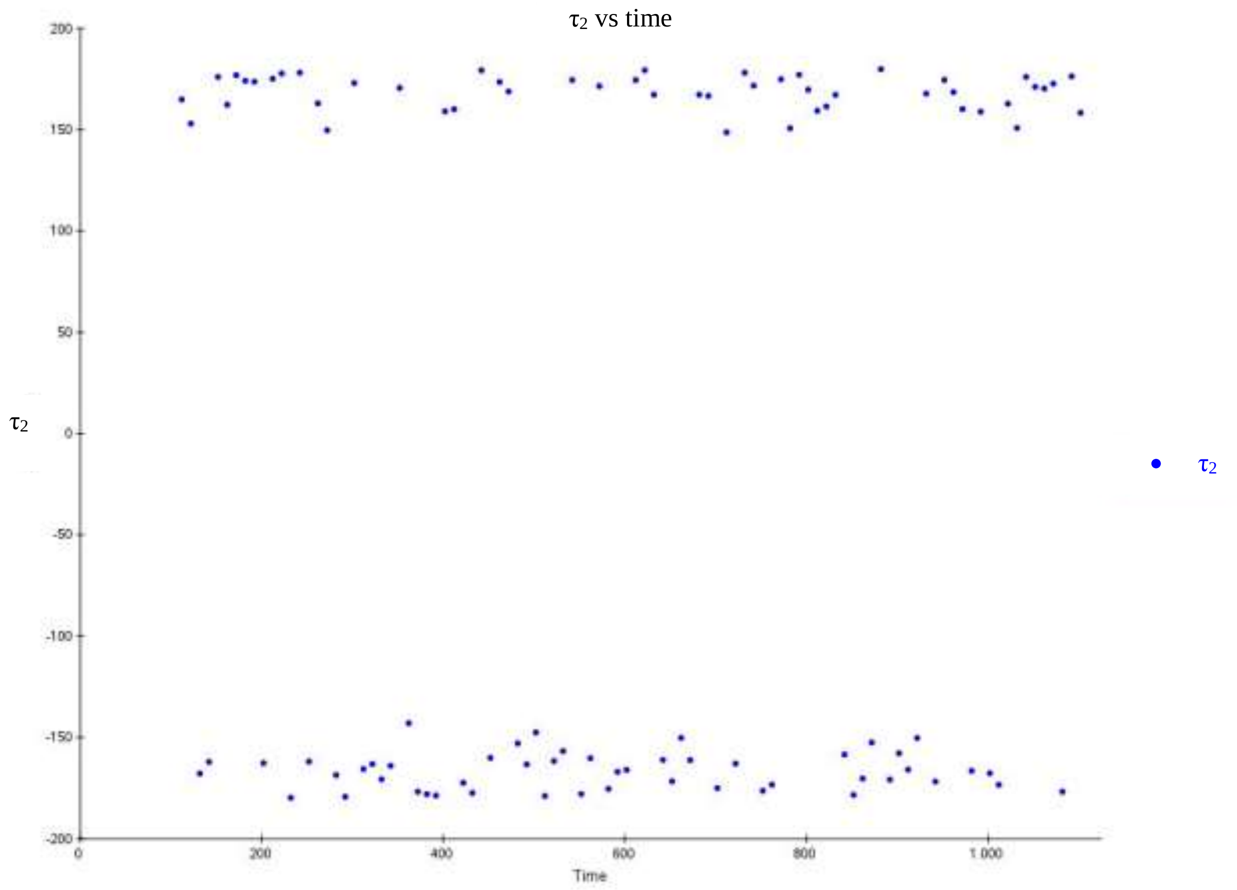

Fig. S2. Dihedral angle τ_1- τ_2 distribution for **7d** during MD simulation (T=600K, ε=4r).

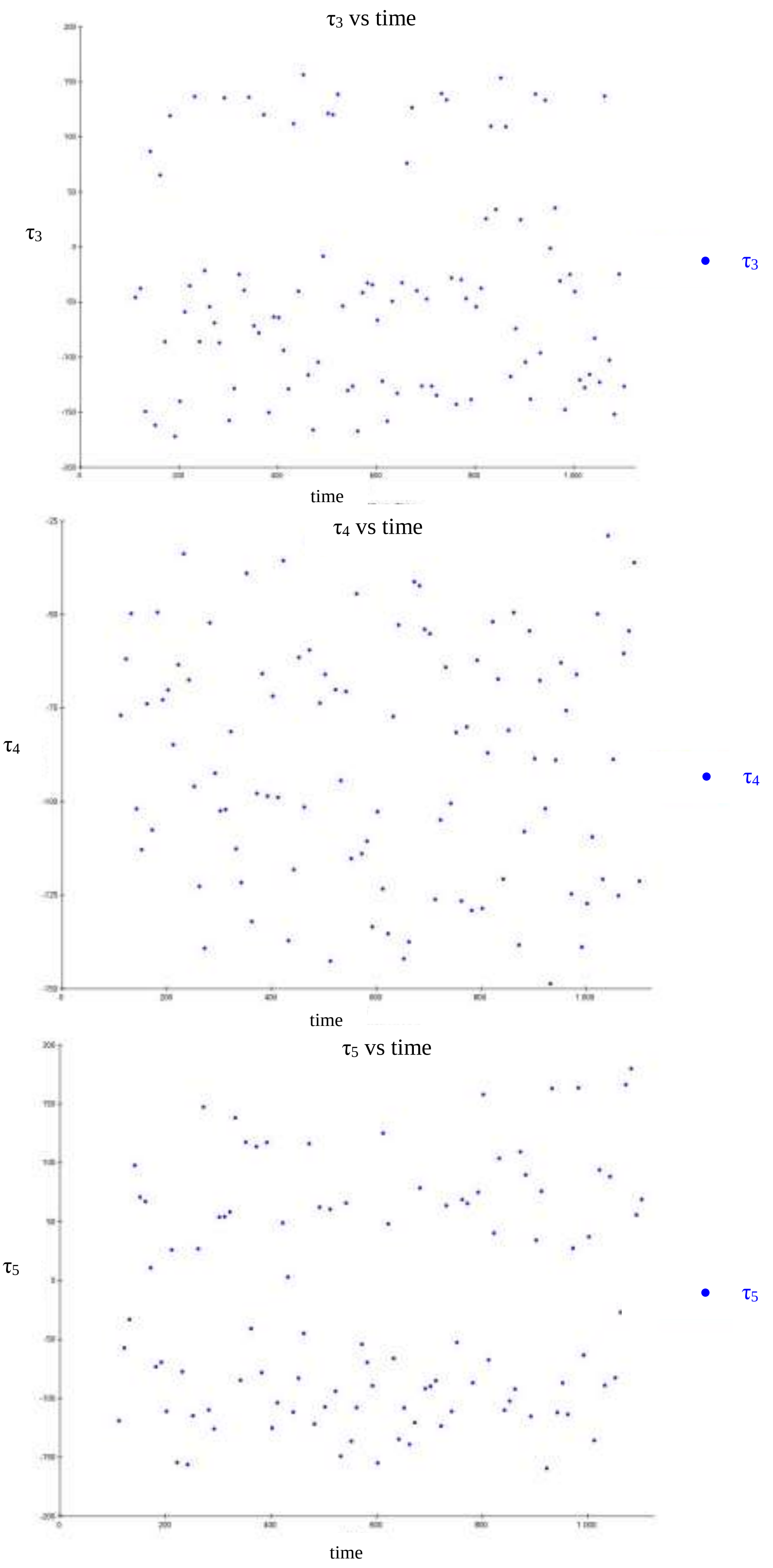

Fig. S3. Dihedral angle τ_3- τ_5 distribution for **7d** during MD simulation (T=600K, ε=4r).

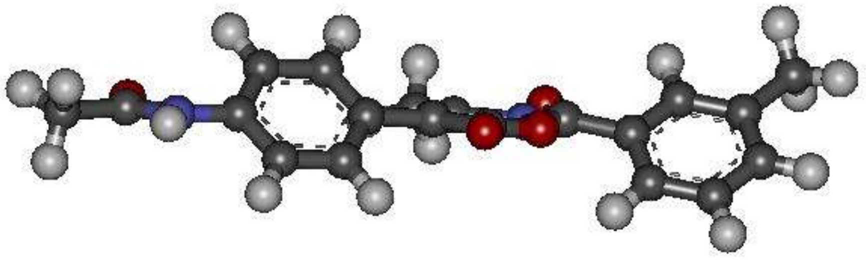

Fig. S4. View of the lowest energy conformer of **7d** as found from MD simulations (T=600K, ε=4r).

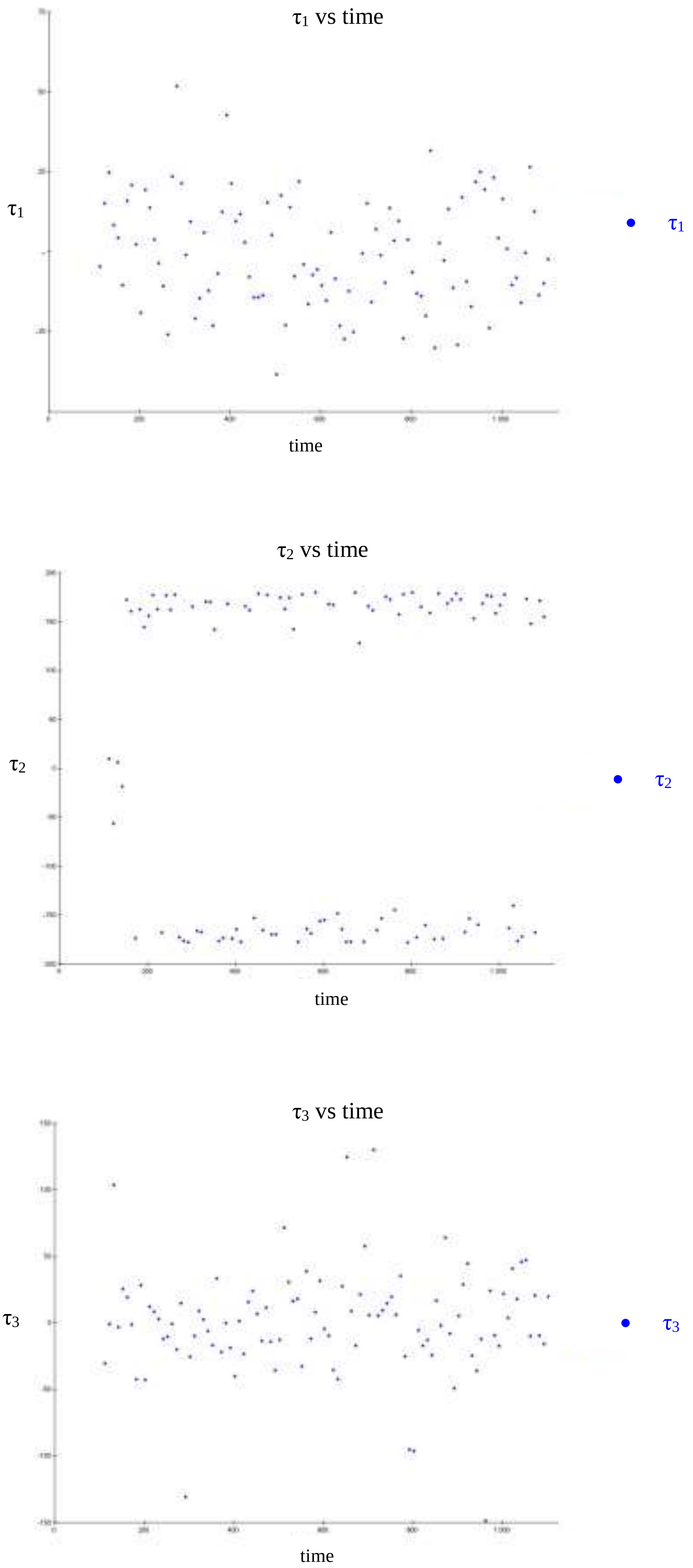

Fig. S5. Dihedral angle τ_1- τ_3 distribution for **8d** during MD simulation (T=600K, ε=4r).

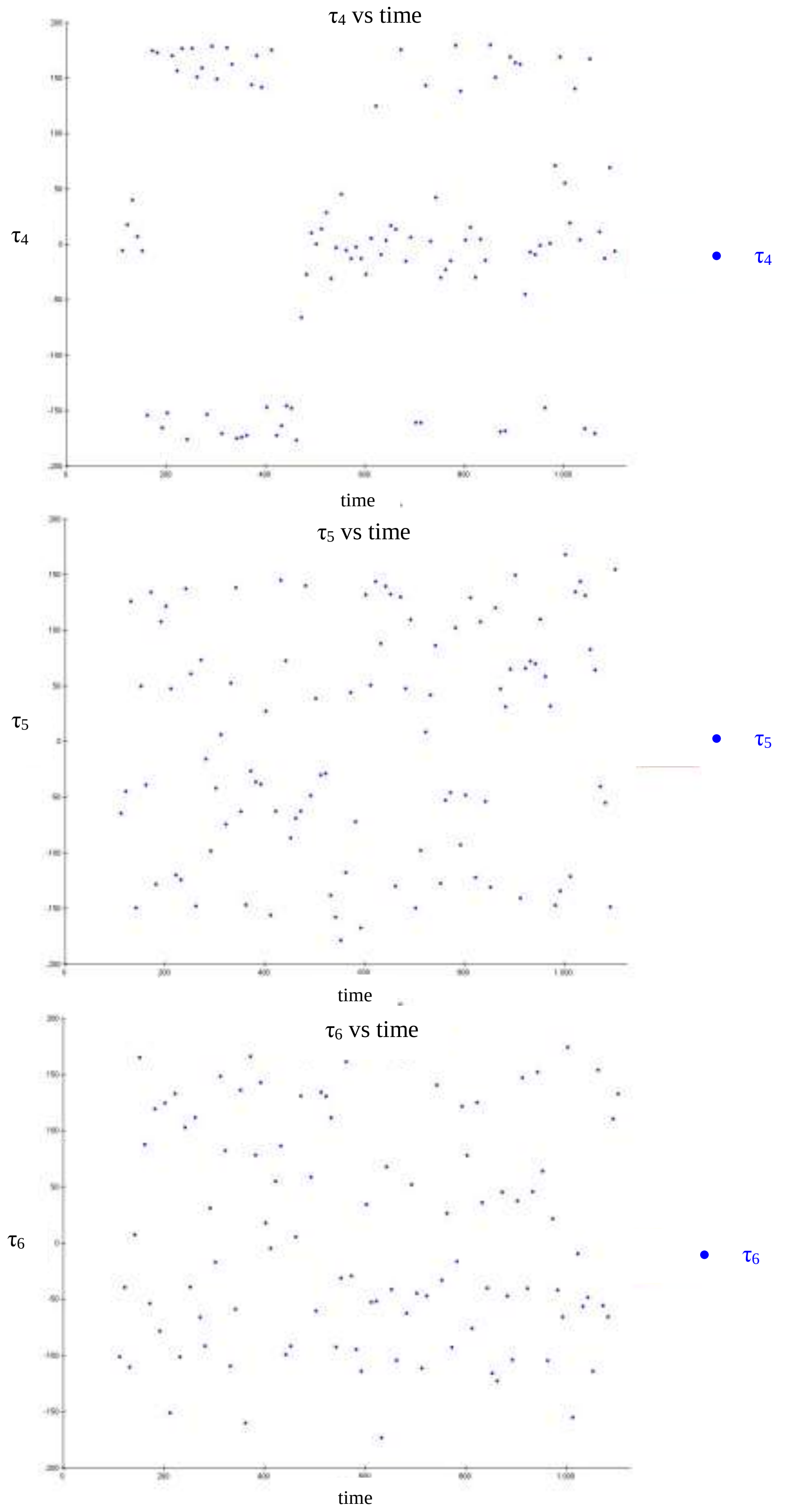

Fig. S6. Dihedral angle τ_4- τ_6 distribution for **8d** during MD simulation (T=600K, ε=4r).

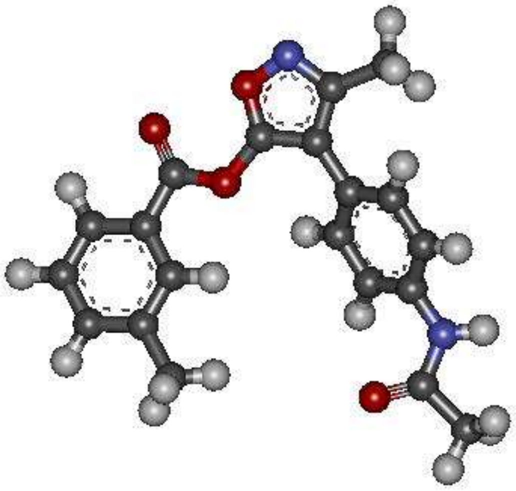

Fig. S7. View of the lowest energy conformer of **8d** as found from MD simulations (T=600K, ε=4r).

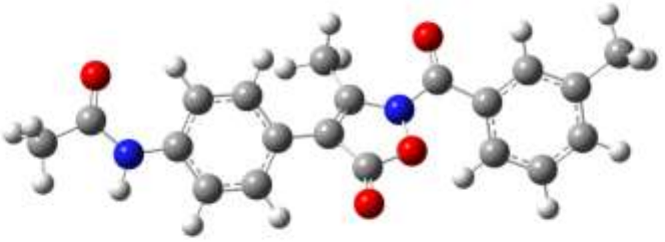

Fig. S8. View of the lowest energy conformer of **7d** as found from QC (B3LYP functional).

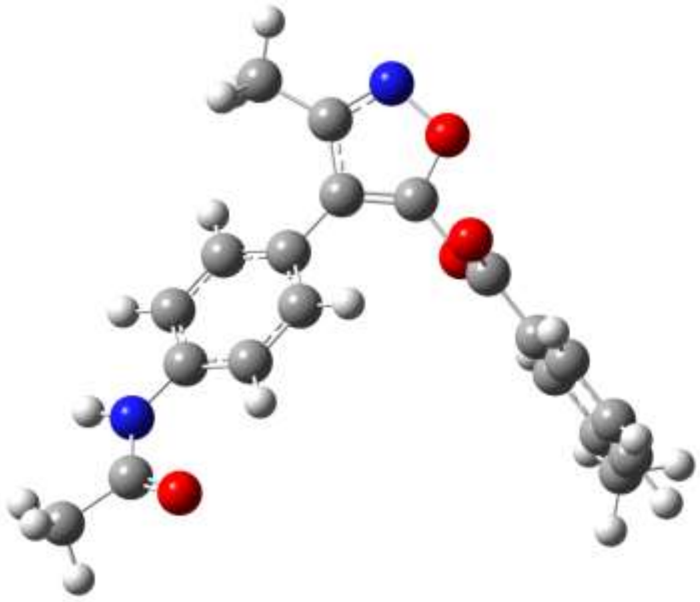

Fig. S9. View of the lowest energy conformer of **8d** as found from QC (B3LYP functional).

Table S1. Elemental analysis

Comp.	Formula (MW)	Anal. Calcd.				Anal. Found		
		C	H	N		C	H	N
**2**	$C_{18}H_{17}NO_2$ (279.33)	77.40	6.13	5.01		77.94	6.15	5.03
**3a**	$C_{16}H_{13}NO_5S$ (331.34)	58.00	3.95	4.23		58.23	3.96	4.25
**3b**	$C_{21}H_{21}NO_6S$ (415.46)	60.71	5.09	3.37		60.95	5.11	3.38
**3c**	$C_{21}H_{22}N_2O_5S$ (414.47)	60.85	5.35	6.76		61.09	5.37	6.78
**4a**	$C_{18}H_{15}NO_3$ (293.32)	73.71	5.15	4.78		74.00	5.17	4.80
**4b**	$C_{14}H_{13}NO_3$ (243.26)	69.12	5.39	5.76		69.39	5.41	5.78
**4c**	$C_{18}H_{15}NO_3$ (293.32)	73.71	5.15	4.78		74.00	5.17	4.80
**4d**	$C_{18}H_{15}NO_3$ (293.32)	73.71	5.15	4.78		74.00	5.17	4.80
**4e**	$C_{18}H_{12}F_3NO_3$ (347.29)	62.25	3.48	4.03		62.50	3.49	4.05
**4f**	$C_{18}H_{15}NO_5S$ (357.38)	60.49	4.23	3.92		60.73	4.25	3.93
**4g**	$C_{18}H_{12}N_2O_3$ (304.30)	71.05	3.97	9.21		71.33	3.98	9.25
**4h**	$C_{18}H_{12}N_2O_3$ (304.30)	71.05	3.97	9.21		71.33	3.98	9.25
**4i**	$C_{22}H_{22}N_2O_4$ (378.42)	69.83	5.86	7.40		70.10	5.88	7.43
**4l**	$C_{22}H_{21}NO_5$ (379.41)	69.64	5.58	3.69		69.91	5.60	3.70
**4m**	$C_{23}H_{17}NO_3$ (355.39)	77.73	4.82	3.94		78.04	4.84	3.95
**4n**	$C_{19}H_{15}NO_3$ (305.33)	74.74	4.95	4.59		75.03	4.97	4.61
**4o**	$C_{18}H_{15}NO_3$ (293.32)	73.71	5.15	4.78		74.00	5.17	4.80
**4p**	$C_{14}H_{13}NO_3$ (243.26)	69.12	5.39	5.76		69.39	5.41	5.78
**4q**	$C_{17}H_{13}NO_3$ (279.29)	73.11	4.69	5.02		73.40	4.71	5.04
**4r**	$C_{13}H_{11}NO_3$ (229.23)	68.11	4.84	6.11		68.38	4.86	6.13

**4s**	$C_{17}H_{12}N_2O_5$ (324.29)	62.96	3.73	8.64		63.21	3.74	8.67
**4t**	$C_{13}H_{10}N_2O_5$ (274.23)	56.94	3.68	10.22		57.16	3.69	10.26
**4u**	$C_{19}H_{16}N_2O_4$ (336.34)	67.85	4.79	8.33		68.12	4.81	8.36
**7a**	$C_{19}H_{17}NO_3$ (307.34)	74.25	5.58	4.56		74.55	5.60	4.58
**7b**	$C_{19}H_{14}N_2O_3$ (318.33)	71.69	4.43	8.80		71.97	4.45	8.83
**7c**	$C_{18}H_{14}N_2O_5$ (338.31)	63.90	4.17	8.28		64.15	4.19	8.31
**7d**	$C_{20}H_{18}N_2O_4$ (350.37)	68.56	5.18	8.00		68.83	5.20	8.03
**7e**	$C_{22}H_{20}N_2O_4$ (376.41)	70.20	5.36	7.44		70.48	5.38	7.47
**8a**	$C_{19}H_{17}NO_3$ (307.34)	74.25	5.58	4.56		74.55	5.60	4.58
**8b**	$C_{19}H_{14}N_2O_3$ (318.33)	71.69	4.43	8.80		71.97	4.45	8.83
**8d**	$C_{20}H_{18}N_2O_4$ (350.37)	68.56	5.18	8.00		68.83	5.20	8.03
**8e**	$C_{22}H_{20}N_2O_4$ (376.41)	70.20	5.36	7.44		70.48	5.38	7.47
**10a**	$C_{10}H_9NO_3$ (191.18)	62.82	4.74	7.33		63.07	4.76	7.36
**10b**	$C_{12}H_{13}NO_3$ (219.24)	65.74	5.98	6.39		66.00	6.00	6.41
**10c**	$C_{15}H_{11}NO_3$ (253.25)	71.14	4.38	5.53		71.42	4.40	5.55
